# Supramolecular Organogels Based on *N*-Benzyl, *N′*-Acylbispidinols

**DOI:** 10.3390/nano9010089

**Published:** 2019-01-11

**Authors:** Alexey V. Medved’ko, Alexander I. Dalinger, Vyacheslav N. Nuriev, Vera S. Semashko, Andrei V. Filatov, Alexander A. Ezhov, Andrei V. Churakov, Judith A. K. Howard, Andrey A. Shiryaev, Alexander E. Baranchikov, Vladimir K. Ivanov, Sergey Z. Vatsadze

**Affiliations:** 1 Faculty of Chemistry, Lomonosov Moscow State University, 119991 Moscow, Russia; lexeym@gmail.com (A.V.M.); dal1995@mail.ru (A.I.D.); nvn@org.chem.msu.ru (V.N.N.); vera-s@yandex.ru (V.S.S.); a.baranchikov@yandex.ru (A.E.B.); 2 Zelinsky Institute of Organic Chemistry, Russian Academy of Sciences, 119991 Moscow, Russia; filatov_andrey_@mail.ru; 3 Faculty of Physics, Lomonosov Moscow State University, 119991 Moscow, Russia; alexander-ezhov@yandex.ru; 4 Topchiev Institute of Petrochemical Synthesis, Russian Academy of Sciences, 119991 Moscow, Russia; 5 Kurnakov Institute of General and Inorganic Chemistry of the Russian Academy of Sciences, 119991 Moscow, Russia; churakov@igic.ras.ru (A.V.C.); van@igic.ras.ru (V.K.I.); 6 Department of Chemistry, University of Durham, Durham DH1 3LE, UK; j.a.k.howard@durham.ac.uk; 7 Frumkin Institute of Physical Chemistry and Electrochemistry, Russian Academy of Sciences, 119071 Moscow, Russia; a_shiryaev@mail.ru; 8 Institute of Geology of Ore Deposits, Petrography, Mineralogy and Geochemistry, Russian Academy of Sciences, 119017 Moscow, Russia; 9 Faculty of Material Science, Lomonosov Moscow State University, 119991 Moscow, Russia

**Keywords:** organic nanomaterials, bispidines, supramolecular gels, SEM, TEM, AFM study, X-ray diffraction, FTIR spectroscopy, ATR spectroscopy, SAXS

## Abstract

The acylation of unsymmetrical *N*-benzylbispidinols in aromatic solvents without an external base led to the formation of supramolecular gels, which possess different thicknesses and degrees of stability depending on the substituents in para-positions of the benzylic group as well as on the nature of the acylating agent and of the solvent used. Structural features of the native gels as well as of their dried forms were studied by complementary techniques including Fourier-transform infrared (FTIR) and attenuated total reflection (ATR) spectroscopy, atomic force microscopy (AFM), transmission electron microscopy (TEM), scanning electron microscopy (SEM), and small-angle X-ray scattering and diffraction (SAXS). Structures of the key crystalline compounds were established by X-ray diffraction. An analysis of the obtained data allowed speculation on the crucial structural and condition factors that governed the gel formation. The most important factors were as follows: (i) absence of base, either external or internal; (ii) presence of HCl; (iii) presence of carbonyl and hydroxyl groups to allow hydrogen bonding; and (iv) presence of two (hetero)aromatic rings at both sides of the molecule. The hydrogen bonding involving amide carbonyl, hydroxyl at position 9, and, very probably, ammonium N-H^+^ and Cl^−^ anion appears to be responsible for the formation of infinite molecular chains required for the first step of gel formation. Subsequent lateral cooperation of molecular chains into fibers occurred, presumably, due to the aromatic π−π-stacking interactions. Supercritical carbon dioxide drying of the organogels gave rise to aerogels with morphologies different from that of air-dried samples.

## 1. Introduction

Positron emission tomography (PET) is one of the widely used molecular imaging methods enabling early and high-resolution diagnostics of various diseases including oncological, neurological and cardiovascular [1,2]. In recent years, there has been growing interest in the field of new radiopharmaceuticals (RPs) with non-conventional PET radionuclides, for example, ^86^Y (14.7 h), ^89^Zr (78.4 h), and ^64^Cu (12.7 h) [3,4,5]. Among others, ^64^Cu is one of the most promising isotopes for PET usage because it possesses the smallest average energy of positrons, and its half-life allows delivery to local clinics [6,7]. Unlike conventional PET radionuclides (^15^O, ^13^N, ^11^C, ^18^F), the incorporation of metal isotopes into RPs requires the use of poly/multifunctional chelating agents that should form covalent bonds with various biomolecules/vectors possessing high specificity and selectivity toward certain targets [5,8,9,10,11]. 3,7-Diazabicyclo [3.3.1]nonanes (hereafter called bispidines) are one of the privileged scaffolds in medicinal chemistry [12] due to the number of advantages, for example, (i) high basicity and solubility in water and/or organic solvents [13,14]; (ii) the ability of diverse functionalization including chiral derivatives [15,16,17,18,19,20]; and (iii) deeply studied conformational features which allow the application of some rigid conformation [21,22,23]. Our recent works have shown the potential of using bispidine scaffolds for the design of inhibitors of serine proteases [24,25]. At the same time, bispidines are well-known ligands with a high affinity to Cu(II) and other bivalent metals [26,27,28,29]. Therefore, we focused on creating new potent ligands for ^64^Cu PET based on a number of unsymmetrically substituted bispidine-9-ols. During our work with ligands for serine proteases, it was necessary to find a way to insert bulky groups into pockets of the active site [24,25]. The acylation reaction is one of the obvious ways to functionalize the secondary amine group in *N*-benzylbispidinols; the resulting *N*-benzyl, *N*′-acylbispidinols allow subsequent transformations like the reduction of the amide group or the removal of the protecting benzylic group followed by the diverse functionalization of the formed secondary amine. In the course of our work, we discovered an interesting phenomenon, namely, the formation of gels during the acylation of *N*-benzylbispidinols in benzene.

Supramolecular gels belong to a broad family of supramolecular materials [30]. Since the beginning of supramolecular gel investigation in the mid-1990s, this area attracts significant interest due to the many exciting and unique features exhibited by this soft matter [31,32,33,34,35,36,37,38,39,40,41]; see also the special issue of the *Beilstein Journal of Organic Chemistry* [42] and the Tetrahedron Symposium-in-Print Number 130 [43]; vol. 256 of *Topics in Current Chemistry* [44]; the special issue of *Gels* (https://www.mdpi.com/journal/gels/special_issues/supramol_gels), and a monograph [45]. The uniqueness of supramolecular gels in manifested in their intrinsic stimuli-responsive nature [46]. Their properties span from high porosity [47] to mechanically robust materials [48]; they can form unexpected composites with polymers [39] and photopatterned materials [49]; they are known for their self-sorting properties [50] and can be used as photophysical materials with unique properties [51,52]. Additionally, they can be exploited as pollutant removers [53] and for programmed cell growth [54].

The synthesis of a family of unsymmetrically *N,N*′-disubstituted bispidine-9-ol derivatives, gelling properties of their HCl salts, and a possible explanation of such behavior will be discussed in this manuscript. The main advantages and features of a new family of organogelators, namely, *N,N*′-disubstituted bispidine-9-ols, may be briefly emphasized as follows. Both (hetero)aromatic groups Ar^1^ and Ar^2^ can be independently varied to allow their introduction into the organogels and, hence, into their aerogel forms, offering a wide diversity of substituents with many useful properties. For example, we began studying in our labs the possibility of obtaining luminescent gels and gels capable of metal ion recognition. The presence of basic nitrogen atoms in *N,N*′-disubstituted bispidine-9-ols allows researchers to study acid/base interactions and their effects on gel stability.

## 2. Materials and Methods

### Synthesis. General Methods

Compounds **1**–**3** were synthesized according to procedures described in [24,55,56].

A solution of acyl halide (1 eq.) in dry benzene was added dropwise to a suspension of bispidine 3 (1 eq.) in dry benzene under vigorous stirring. In most cases, a gel-like substance started to form after the first several drops of the acyl halide solution. Then the mixture was refluxed under vigorous stirring for 3.5 hours. To obtain the hydrochloride product, the resulting gelatinous mass (precipitate or colloidal solution) was centrifuged for 10–15 min (6000 rpm) or filtered on a Schott filter (por.40) and then air dried with subsequent drying on a rotary evaporator. The free base was isolated from the aqueous solution of the formed solid hydrochloride by treatment with sodium bicarbonate (3 eq.) and characterized (Supplementary Materials, page 3–10). To study the native benzenogels, the reaction mixture was allowed to cool down to room temperature (see below).

Supercritical carbon dioxide drying was performed using a home-made system (volumes 5 mL or 80 mL;, for details, see [57,58]). As per typical experiments, the working pressure and temperature were 80–90 bar and 40–50 °C, respectively.

Small-angle X-ray scattering and diffraction (SAXS). SAXS patterns were acquired using a dedicated small-angle diffractometer SAXSess (Anton Paar, Graz, Austria) employing monochromatic Cu-*K*α1 radiation (1.5412 A). The samples were placed into a standard glass capillary 1.5 mm in diameter and sealed. The measurements were performed in vacuum; scattered radiation was recorded using 2D imaging plates. 

Fourier-transform infrared (FTIR) spectra were acquired using a SpectrumOne spectrometer coupled with an AutoImage microscope (Perkin Elmer, Walthan, MA USA). The samples were placed on gold mirror and the spectra represented the superposition of absorption (dominant) and reflectance components.

Atomic force microscopy (AFM). AFM measurements were made using a scanning probe microscope NTEGRA Prima (NT-MDT, Moscow, Russian Federation). Semi-contact mode was used. The amplitude of the “free air” probe oscillations was from 20 to 25 nm (peak-to-peak). High-resolution non-contact/semi-contact silicon AFM probes of the “Golden” NSG01 series (NT-MDT) were used. Topography, feedback error signal, and phase difference were registered simultaneously for the samples. The samples were prepared by drying the solution on silicon wafers.

Transmission electron microscopy (TEM). TEM images were obtained using a transmission electron microscope LEO912 AB OMEGA (Carl Zeiss, Oberkochen, Germany) operating at 100 kV voltage. Samples were prepared by drying the solution droplets placed on copper grids coated by a Formvar film.

Scanning electron microscopy (SEM). SEM images were obtained using a Carl Zeiss NVision 40 (Carl Zeiss, Oberkochen, Germany) workstation at 1 kV accelerating voltages using a secondary electron (SE2) detector. The preparation of the samples was the same as for the AFM measurements.

X-Ray. Experimental intensities were measured using Bruker SMART 1K (for **4cb** and **2c**) and Bruker SMART APEX II (Brucker, Madison, WI, USA) diffractometers (for **4cb**, **3c**, **1b**, **4da**, and 5,7-dimethyl-1,3-diazaadamantan-6-one) using graphite monochromatized Mo-*K*α radiation (λ = 0.71073 Å) in ω-scan mode. Absorption corrections based on measurements of equivalent reflections were applied. The structures were solved by direct methods and refined by full matrix least-squares on F^2^ with anisotropic thermal parameters for all non-hydrogen atoms (except the solvent benzene molecule in **4da**) [59]. In **1b** and **4da**, all hydrogen atoms were placed in calculated positions and refined using a riding model. As for structures **4cb**, **2c**, **3a**, and **1b**, all protons were added geometrically and refined using a riding model, whereas all hydroxy- and amino-hydrogens were located from difference Fourier synthesis and refined isotropically. Finally, in **3c** and 5,7-dimethyl-1,3-diazaadamantan-6-one, all protons were found from the difference map and their positional and thermal parameters were refined. The crystals of **4cb** and **1b** were racemically twinned with domain ratios 0.79/0.21 and 0.76/0.24, respectively. X-ray diffraction studies were performed at the Centre of Shared Equipment of the Institute of General and Inorganic Chemistry of the Russian Academy of Sciences (IGIC RAS). The crystallographic data have been deposited with the Cambridge Crystallographic Data Centre (CCDC) as supplementary publications (for CCDC numbers, see Table S3). This information may be obtained free of charge from the Cambridge Crystallographic Data Centre website (www.ccdc.cam.ac.uk/data_request/cif).

## 3. Results

**Scheme 1:**
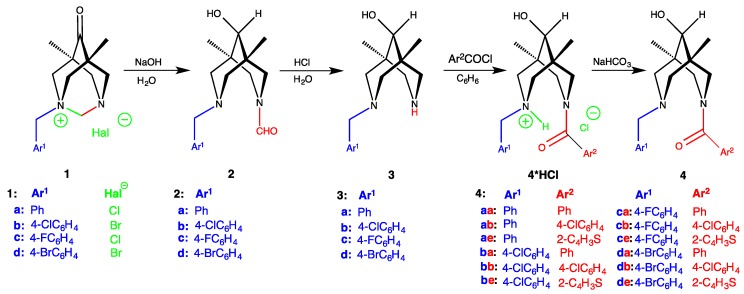
Synthetic routes to the target amides **4**.

Compounds **1**–**3** were prepared according to protocols from [24,55,56]: the stereoselective ring opening in salts **1** leads to unsymmetrically substituted bispidinols **2**; the deformylation of the latter gives rise to secondary amino alcohols **3** (Scheme 1).

The next step, the acylation of the secondary amine group in **3**, is straightforward due to the presence in the same molecule of a tertiary amine group which would work as an internal base to accept released HCl. It is expected that the resulting salts 4*HCl should precipitate from the reaction mixture making the isolation easier. Indeed, the tertiary amine group worked as the internal base during the acylation, but unexpectedly, instead of the precipitation, the acylation of molecules **3** in benzene in most cases (except for **4aa**, **4ba**, and **4ca**) led to the formation of opaque to almost transparent gel-like materials—very viscous substances with the eventual presence of crystalline admixtures (see Figures S1–S7). Similar results were obtained when we used nitrobenzene, ethoxybenzene, or mesitylene (in this case, the least stable gels if any were produced) instead of benzene for the synthesis of 4ab*HCl and 4ae*HCl.

The target amides **4** were finally obtained by gentle base treatment of the salts 4*HCl (Supplementary Materials, page 3–6). It was found that the choice of the base is crucial to obtaining the amides **4**: using a strong base like alkali leads to de-acylation instead of the formation of target amino-amides. Presumably, this is due to the spatial proximity of an amino lone pair and an amide function. Finally, we were able to isolate and characterize 12 new amino-amides **4** which differed by substituents Ar^1^ and Ar^2^ (see Scheme 1).

In order to understand the structural features of the gels with general formulae benzene@4*HCl, several physical-chemical methods providing information about various structural levels of supramolecular gels [30,31,32,60,61] were applied: on the molecular scale (X-ray diffraction, IR spectroscopy), nanoscale (SAXS, SEM, TEM, AFM), and macroscopic scale (IR and NMR spectroscopy, SAXS, DLS, DSC, POM, rheology). Some of the applied methods failed to provide useful information. For example, we were unable to obtain reasonable results from the NMR-spectroscopic titration of solution of **3** by acyl chloride solution since all signals appeared to be quite broad. Rheological studies applied to sample benzene@4ce*HCl showed that this was indeed a gel [62], but no quantitative information emerged (Tables S1 and S2, Figures S8 and S9).

**Scheme 2:**
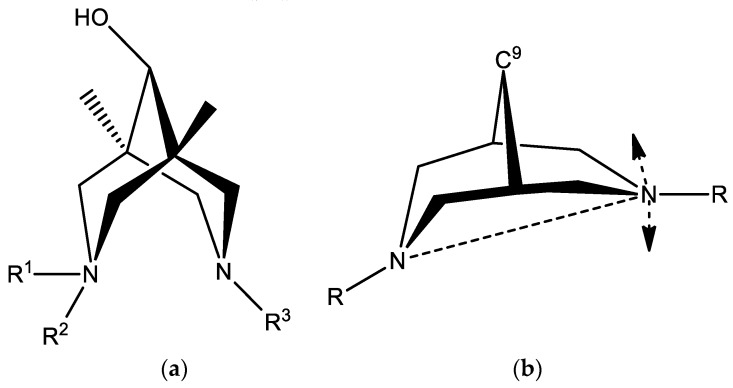
(**a**) Chemical connectivity scheme for structurally characterized samples. (**b**) Distances and deviations.

In order to understand possible mechanisms of the intermolecular interactions that would lead to gel formation, we applied the single-crystal X-ray technique to those samples that were crystalline. The chemical connectivity scheme for structurally characterized samples **4cb**, **2c**, **3a**, **3c**, and 4da*HCl is presented in Scheme 2a, whereas their molecular structures are shown in Figures S10–S17. Selected geometrical features are listed in Table 1. The main geometrical parameters of the bispidine skeleton in all studied compounds are close to the values reported and discussed for numerous bispidines and their salts in [63] and references cited therein.

In all presented cases, both piperidine cycles of bicyclic skeleton adopt a chair conformation with involuntarily shortened N…N separations (see Table 1). In all five bispidine structures, benzyl-substituted nitrogen atoms (NBz) are almost tetrahedral with the sum of C-NBz-C angles ranging from 330.0 to 332.1°. Benzyl substituents always occupy axial positions with respect to six-membered piperidine cycles (*exo-* to the concave surface of an eight-membered cycle). Of interest, hydroxyl groups at C9 positions are oriented in the same direction as the benzyl substituents. As expected, amido nitrogen atoms in **2c**, **3c**, and 4da*HCl are almost planar due to their sp^2^ nature and do not participate in hydrogen bonding. In contrast, secondary amine N atoms in **3a** and **3c** are tetrahedral with the sum of angles nearly equal to 325°. Their hydrogen atoms are *endo*-oriented due to intramolecular hydrogen bonding.

**Figure 1:**
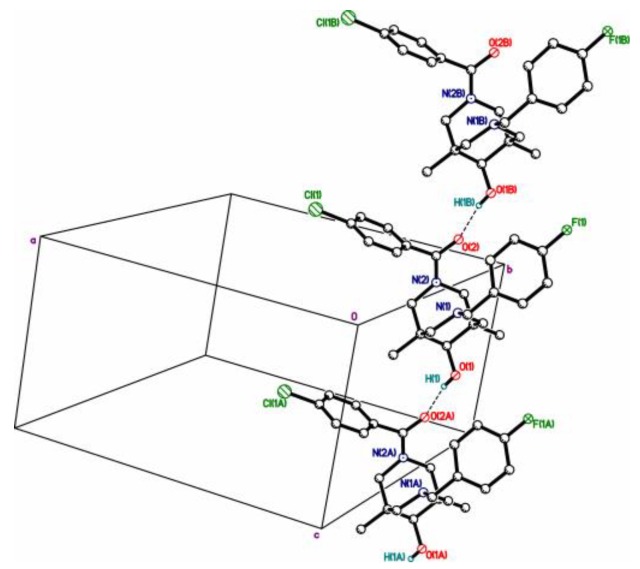
Hydrogen-bonded chains in structure **4cb**.

The most intriguing feature of the bispidine derivatives is the conformational flexibility of both piperidine cycles. In general, they can adopt chair-chair, chair-boat, or boat-boat conformations [22,64]. Search in the Cambridge Structural Database (CSD, ver. 5.39, Feb 2018) [65] for structures of neutral organic flexible bispidine derivatives with sp^3^- hybridized carbon atoms within an eight-membered cycle produced 121 entries (136 molecules). Several additional structures with additional fused rigid cycles were rejected. Examination of the nitrogen–nitrogen separation distribution reveals that the widely used N…N distance is an inappropriate parameter for conformational analysis of bispidines. In close proximity to the intermediate point (half-chair arrangement with one almost planar N atom), significant shifts of the nitrogen atom (perpendicular to the main plane of the cycle) do not lead to noticeable changes in N…N distances (Scheme 2b). Therefore, the mutual distribution of C9…N distances was analyzed (Figure 1). Regions corresponding to chair-chair and chair-boat conformations are clearly separated on this scatterplot. The chair-chair arrangement dominates: 87 vs. 49 cases. Interestingly, in the crystalline state, the boat-boat conformation is absent. Thus, other criteria should be used to distinguish chair-chair and chair-boat conformations: namely, the absolute value of the difference between C9…N distances (Figure S18-S19). The approximate value 0.17 Å may be used as a threshold between these conformations.

In structures **4cb**, **3c**, and **4da**, one of the two nitrogen atoms bears a H atom available for hydrogen bonding. It is of interest that, in all cases, these atoms are involved in strong intramolecular hydrogen bonding NH…N. Obviously, such hydrogen bonding is assisted by conformational preorientation of the bispidine scaffold.

**Figure 2:**
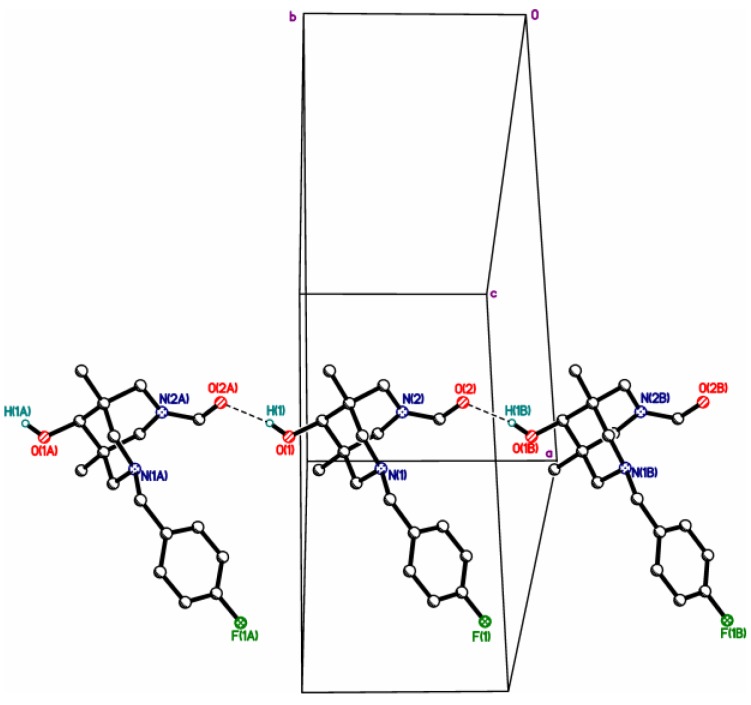
Hydrogen-bonded chains in structure **2c**.

**Figure 3:**
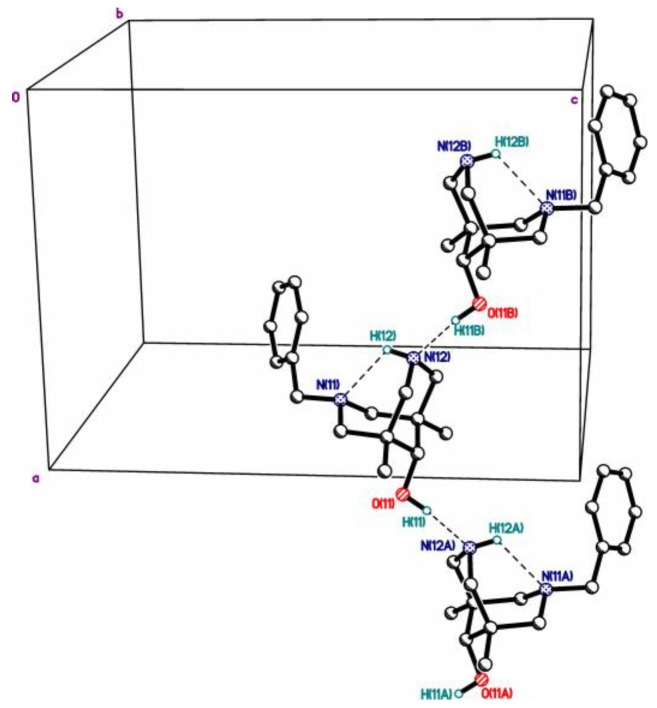
Hydrogen-bonded chains in structure **3a**.

**Figure 4:**
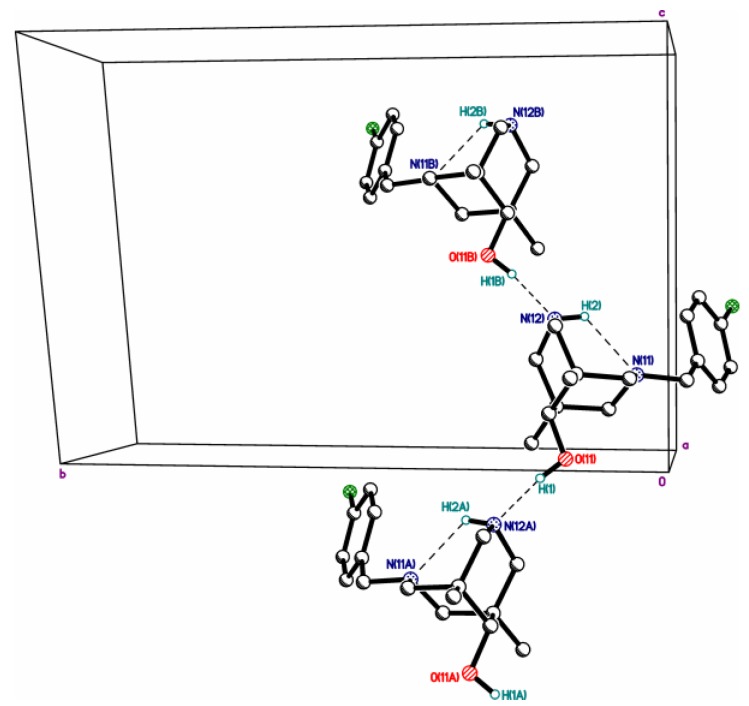
Hydrogen-bonded chains in structure **3c.**

**Figure 5:**
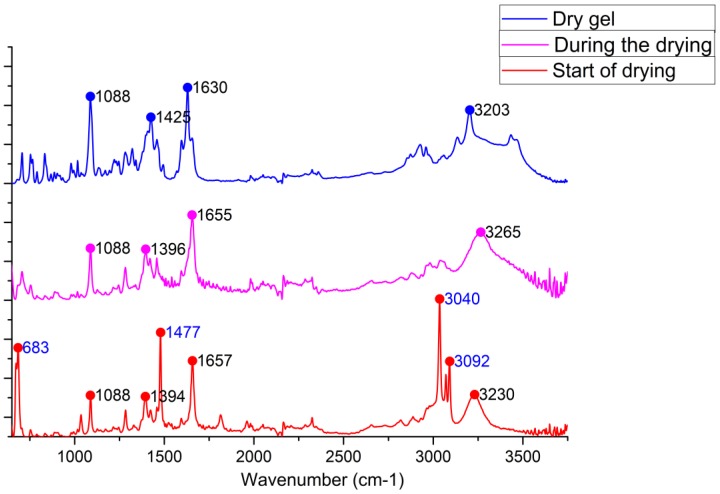
The changes in ATR spectra of **4ab** during solvent removal. Benzene peaks are shown in blue. See text for details.

**Figure 6:**
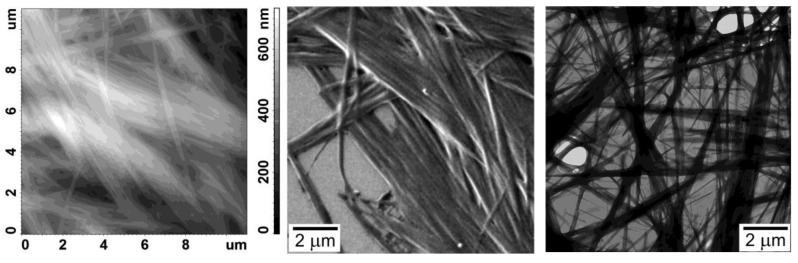
AFM, SEM, and TEM images for the dried gel benzene@4ce*HCl.

Thus, all five bispidinols contain just one “active” hydroxyl hydrogen atom suitable for the formation of intermolecular hydrogen bonds. Additionally, all these molecules contain several acceptors of hydrogen bond at the opposite side, promoting the formation of the H-bonded chains. Actually, such chains formed by OH…O=C or OH…N bonds were observed in the structures of the neutral bispidinols **4cb**, **2c**, **3a**, and **3c** (Figure 2, Figure 3, Figure 4, Figure 5 and Figure 6). In contrast, the structure of salt 4da*HCl contains an insular 0D hydrogen-bonded motif due to the OH…Cl hydrogen bond (Figure S14).

It should be noted that **3c** is isostructural with its chlorine analogue LEFFIN (**3b**) [66].

Structure **1c** comprises both ketone and its bis-diol forms. The molecular structures of the diazaadamantane derivatives **1c** and **1c+H_2_O** are shown in Figures S15 and S17. The main geometrical features of the diazaadamantane scaffold in both compounds are close to those found for hydrochloride of the parent compound [63]. Notably, this is only the third known example of the cocrystallization of ketone and its hydrate [67,68]. Surprisingly, the conformations of both forms are very close to each other. In **1c+H_2_O**, the diol form is stabilized by hydrogen bonding with the adjacent chlorine anions and water molecules (Figure S15). This hydrogen-bonded system is finite, and this structure represents the 0D network. It should be noted that the bezylation of one of the nitrogens in 5,7-dimethyl-1,3-diazaadamantan-6-one (X-ray-derived structure shown in Figure S16) leads to the inequivalence of CH_2_-N bonds in both **1c** and **1c+H_2_O**: the one connected to N+ became longer (1.546/1.565 Å) than the initial one in the starting diazaadamantanone (1.466 Å), whereas the second bond became slightly shorter (1.422/1.437 Å). This is a consequence of the n_N_ – σ*_N–C_ anomeric type electronic interaction already mentioned in [69].

The infinite hydrogen-bonded chains in solid-state structures is a common motif for **2c, 3c, 4cb**. In the case of the amine **3c**, the secondary nitrogen lone pair serves as a hydrogen bond acceptor, whereas in the amides **2c** and **4cb**, carbonyl oxygens play this role. The structure of the salt 4da*HCl differs dramatically, since two types of intermolecular hydrogen bonds can be distinguished within it. The first one binds the protonated tertiary amine hydrogen and carbonyl oxygen of the adjacent centro-symmetrically linked molecule forming the dimers. Each organic molecule is also linked to the chloride anion by the second intermolecular hydrogen bond involving the hydroxyl hydrogen at position 9, which serves as a hydrogen bond donor as in **2c**, **3c**, and **4cb**.

On the basis of these findings for the dried materials, we can tentatively suggest that the main structural motif that links molecules into the 1D supramolecular polymer within the supramolecular gel is the hydrogen bond between the hydroxyl at position 9 and the hydrogen bond donor, which can be either a halogenide anion or a carbonyl oxygen atom. The involvement of the hydroxyl and carbonyl groups in hydrogen bonding is confirmed by FTIR spectra of the native gels benzene@4ce*HCl and benzene@4de*HCl (Figure S25).

**Figure 7:**
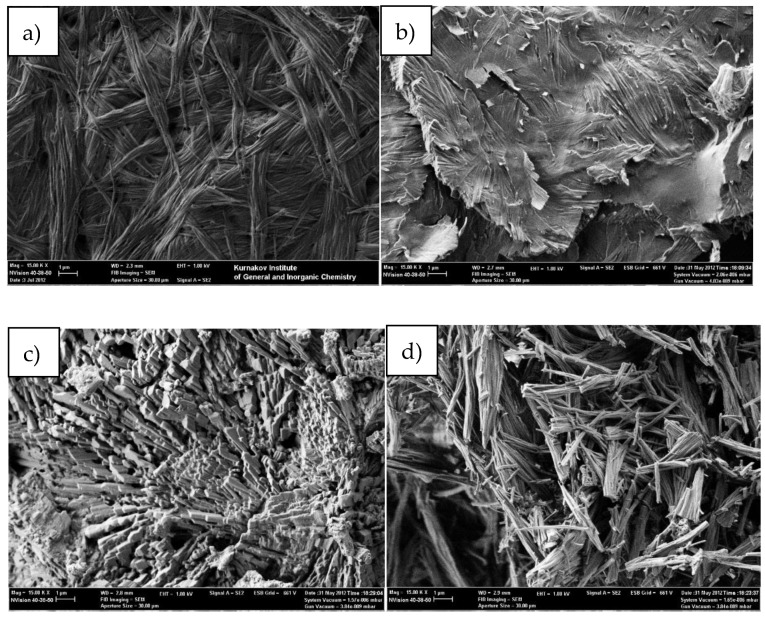
SEM micrographs of dry gel samples obtained by different solvent removal methods from benzene@4cb*HCl: (**a**) bulk xerogel; (**b**) gel coated on the glass surface; (**c**) liquid CO_2_ washing; (**d**) sc-CO_2_ washing. Scale bar is 1 micrometer.

It should be stressed here that upon the removal of benzene and ethoxybenzene, the gel structure undergoes irreversible changes clearly seen in the carbonyl and hydroxyl/NH regions of the IR spectra (Figure 7). These changes indicate heavy structural rearrangement of the gel which leads to the insolubility of the dried sample even in hot benzene, which was attempted in order to prepare a gel from the solid 4*HCl. This can be explained as follows: the removal of the solvent moves previously loosely packed molecular chains into close proximity with the formation of stronger interchain attractive interactions; another explanation can be found in the changes in intermolecular H-bonding (see below in Discussion).

**Figure 8:**
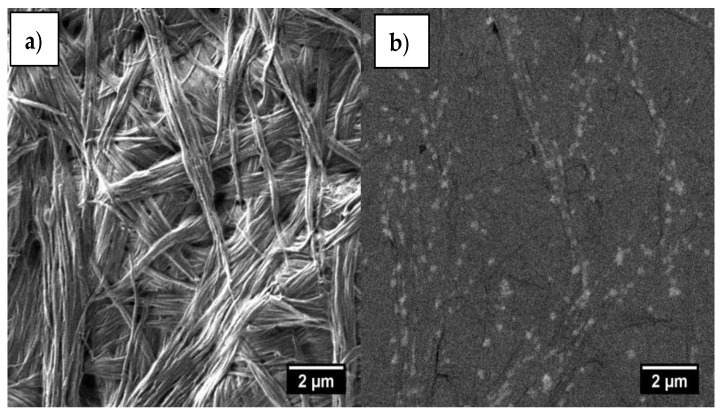
SEM micrographs of the benzene@4cb*HCl xerogel sample obtained for the same area by (**a**) SE2 and (**b**) BSE detectors. Scale bar is 2 μm.

The xerogel organization at the nanoscale level of type 4*HCl obtained by direct benzene evaporation was studied by AFM, SEM, and TEM (Figure 8, Figures S20–S24). The data clearly show the existence of long and thin nanofibers as a main structural motif of the xerogels, which is typical for supramolecular gels [32]. In some cases, the nano-crystalline additives are observed. In some extreme cases, the width of the nanofibers is less than 100 nm.

The sample of benzenogel benzene@4cb*HCl was subjected to different solvent removal procedures.

**Figure 9:**
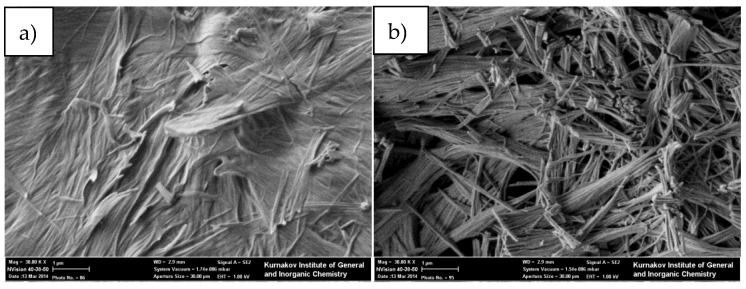
SEM micrographs of dry gel samples obtained by different solvent removal methods from benzene@4ce*HCl: (**a**) direct air-drying; (**b**) sc-CO_2_ drying. Scale bar is 1 μm.

The drying regime drastically affects the microstructure of the gels. Figure 9 shows scanning electron microscopy images of benzene@4cb*HCl gels dried under different conditions. Drying under ambient conditions results in dense structures ranging from strongly interwoven to partially conglutinated fiber-like particles (Figure 9a,b). Drying in supercritical CO_2_ resulted in a loose structure possessing a lower aggregation degree of individual fibers (Figure 9d). Presumably the difference is due to the contribution of capillary forces. Drying under ambient conditions (xerogels) results in a strong coalescence of the particles upon the removal of liquid benzene and a movement of menisci into the sample monolith. The supercritical drying almost levels the effects from the capillary forces and results in loose unaggregated monoliths. The chemical composition of the benzene@4cb*HCl xerogel sample is in agreement with its chemical structure: indeed, the sample contains Cl and F.

Unusual structures were formed upon washing the gel using liquid CO_2_ with subsequent solvent removal (Figure 9c). The gel’s fiber-like structure looks almost disrupted, and the sample consists of strongly coalesced particles with a crystal-like shape; some particles are even faceted. Such an appearance may be due to the substance’s partial dissolution in liquid CO_2_ followed by re-crystallization and fast desorption of CO_2_ from the sample surface, resulting in the disruption of fibers and the formation of dendrite-like particles. The same observations were found for samples prepared from ethoxebenzene (Figure S24)

**Figure 10:**
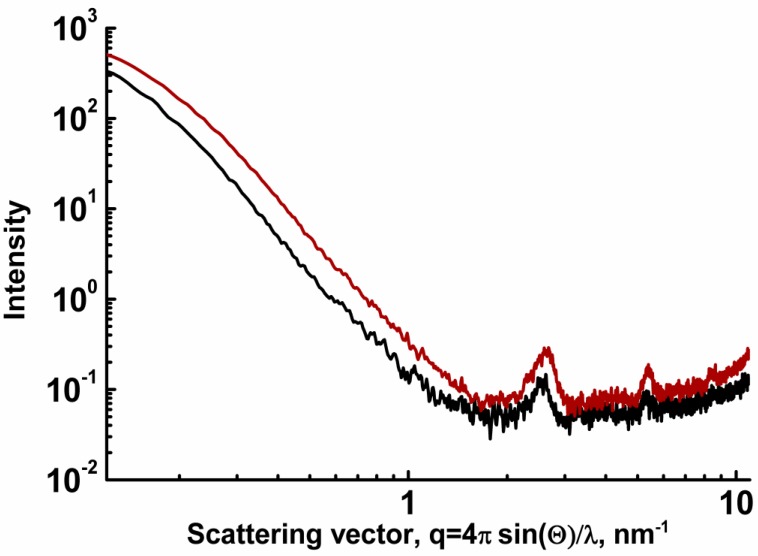
Small-angle X-ray scattering patterns of samples benzene@4ce*HCl (red) and benzene@4de*HCl (black).

An investigation of the xerogel sample using a back-scattered electron detector (BSE-mode) (Figure 10) reveals inclusions of a secondary phase with a density notably higher than that of the matrix. This finding is in agreement with POM data (Figure S7). This can be explained by the formation of some nano-crystalline domains within the amorphous gel fibers.

Similar differences were observed upon drying the benzene@4ce*HCl sample under ambient conditions and in supercritical CO_2_ (Figure 9).

The nanoscale structure of the gels leads to a highly heterogeneous spatial distribution of electron density reflected by intense small-angle X-ray scattering (SAXS) caused by scatterers larger than 60 nm. The samples benzene@4ce*HCl and benzene@4de*HCl also possess crystallographic ordering with interlayer spacings of 2.47, 1.16, and 0.75 nm (benzene@4ce*HCl) and 2.4+2.7 (double peak), 1.16, and 0.75 nm (benzene@4de*HCl) (Figure 10).

The close similarity in scattering patterns of the two different samples undoubtedly indicates the similar nano- and macrostructural organization of benzenogels based on *N*-benzyl, *N’*-acylbispidinols. The existence of the crystallographic ordering may be a result of the ordering of gelator molecules within chains, fibers, and bundles. It can also point to the presence of nano-crystalline domains within the whole gel-like material.

## 4. Discussion

In previous sections we have shown that the acylation of secondary amines **3** in benzene by acyl chlorides led to the formation of supramolecular gels of general formulae benzene@4*HCl. Before we start to speculate on the structural features of new materials, some additional information and data should be taken into account:

- the gels are formed only during the acylation of secondary amines of *N*-benzylbispidine-9-ols, but not upon purging the HCl gas through the solution/suspension of isolated amino-amides **4** in benzene;
- during the removal of benzene from the gel, the latter undergoes some irreversible structural rearrangements which lead to the further insolubility of dried material in benzene;
- the gels are formed neither in the presence of the external base (e.g., triethylamine), nor from the chloroanhydrides of picolinic acids (beta or gamma) or pyrazole-containing acid chlorides;
- the removal of the chloride anion by the action of aqueous silver nitrate destroys the supramolecular gel;
- the gels are not formed upon alkylation (e.g., by benzylchloride);
- the gels are formed only from aromatic (benzoic, para-chlorobenzoic acid) or heteroaromatic (2-thiophencarboxylic acid) acid chlorides, but not with the use of aliphatic reagents (acetylchloride, chloroanhydride, or cyclopropanecarboxylic acid);
- the gels are formed also in nitrobenzene, ethoxybenzene, and mesitylene at elevated temperatures (nearly 100 °C), but their stability depends on the substituents at acylating agents (SI pp. 6–9).

All these data indicate that for the formation of supramolecular gels in our systems, one needs the following: (i) proton (in the form of a protonated tertially amino group, which was found in the crystal of 4da*HCl); (ii) chloride anion (obviously, to form hydrogen bonds with N-H+, as in 4da*HCl, or O-H, as in **1c+H_2_O**); (iii) carbonyl group (obviously, to form infinite chains via hydrogen bonds with O-H, like those found in the crystal structure of **4cd** or **2c**); (iv) two aromatic substituents at both nitrogen atoms of the bispidine scaffold (presumably, to form the lateral intermolecular bonding via π−π-stacking interactions).

**Scheme 3:**
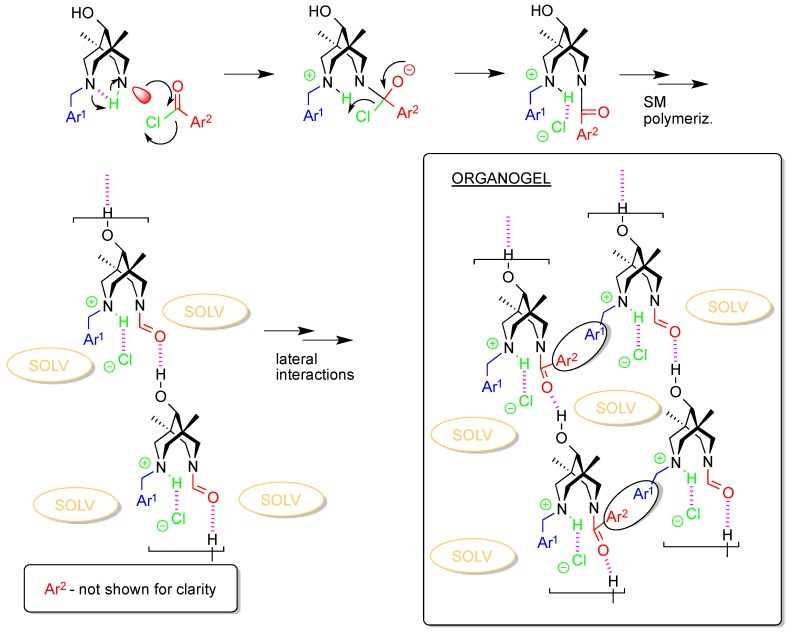
Schematic representation of a possible method of gel formation.

The combination of all features mentioned above allows us to formulate the following mechanism of gel formation upon acylation of amines **3** (Scheme 3). The possible participation of H-bonded supramolecular polymers like those found in the crystals of **3a** or **3c** is not discussed here for simplicity, although they may play some role in the beginning of the process.

Scheme 3 accounts for all of the factors and features mentioned above. Indeed, all the required items are presented in the scheme and all play crucial roles for the gel formation process. At this moment, we lack enough data to reveal the mechanism for the aggregation of species of molecular size, schematically shown as a result of supramolecular polymerization that includes both the formation of H-bonds and π−π-stacks. The role of the solvent in these processes is also unclear, but we can assume the importance of the donor/acceptor properties of the aromatic solvent as well as its steric demands. Indeed, in the case of nitrobenzene, the strongest gel is formed when the π-donor thiophene acid chloride is applied. On the other hand, the sterically demanding mesitylene forms least stable gels among this row of solvents: nitrobenzene, mesitylene, benzene, and ethoxybenzene.

**Scheme 4:**
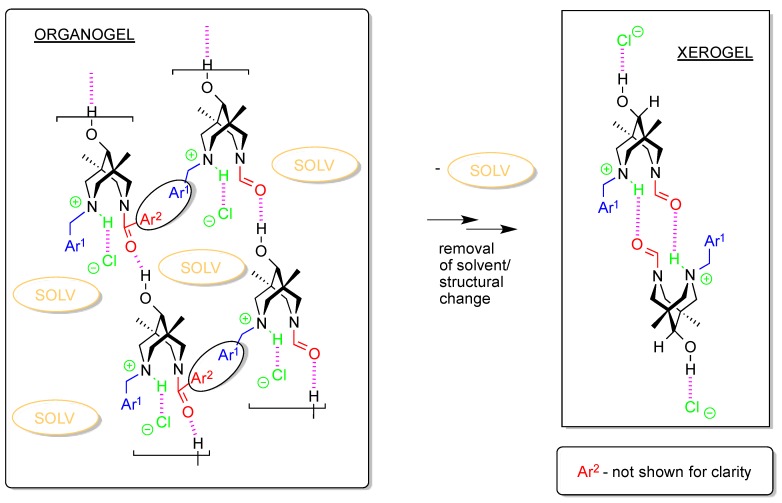
Schematic representation of the structural changes upon solvent removal from the benzenogel.

At the same time, assuming that the organogel building block shown in Scheme 3 indeed appeared during the gel formation, we can explain the changes in gel structure during the solvent removal (see Scheme 4). At the molecular/supramolecular level, one expects the change in the main H-bonded motif from polymeric chain to discreet dimers of type [4da*HCl]_2_. This explains the evolution of the IR spectra during the solvent evaporation: both OH/NH and C=O regions display remarkable changes in the corresponding vibrations (see above). Obviously, such changes will affect the nano/macro level of the gel organization, and different solvent removal methods will result in different morphologies of the final solids.

## 5. Conclusions

In conclusion, we have discovered a new family of low molecular weight gelators (LMWGs) based on the HCl salts of unsymmetrical *N*-benzyl, *N’*-acylbispidinols. The first example of the aerogel preparation from the supramolecular gel is reported in this manuscript. The scope and limitations of the new class of LMWGs is the subject of our current studies. The investigation of copper and zirconium complexes of new ligands and their gel forms for possible PET applications is also on our agenda.

## Figures and Tables

**Table 1:** Selected geometric parameters for bispidines **4cb**, **2c**, **3a**, **3c**, **4da**.

	4cb	2c	3a	3c	4da*HCl
**R1, R2** **R3**	p-FC_6_H_4_CH_2_-, —p-ClC6H4C(=O)-	p-FC_6_H_4_CH_2_-, —-CH(=O)	C_6_H_4_CH_2_-, —H	p-FC_6_H_4_CH_2_-, —H	p-BrC_6_H_4_CH_2_-, HC6H4C(=O)-
**C-OH**	1.423(3)	1.472(6)*	1.4195(12)	1.4178(12)	1.416(12)
av N-Cendo **	1.469(3)	1.458(3)	1.4695(15)	1.4691(14)	1.458(14)
NBz-C **	1.465(3)	1.471(3)	1.4653(14)	1.4658(13)	1.505(13)
N-CO **	1.339(3)	1.331(3)	—	—	1.375(13)
**Σ** C-NBz-C ***	330.3	330.0	331.4	331.9	332.1
**Σ** C-Namid-C ***	359.4	359.5	—	—	353.7
Σ ang NH ***	—	—	325.0	325.8	—
X, O…X ****	=O, 2.784(3)	=O, 2.665(5)	R2HN, 2.7250(12)	R2HN, 2.7157(12)	Cl-, 3.049(10)
N…Nintra *****	2.825(3)	2.830(4)	2.8372(13)	2.8309(13)	2.790(14)

* for major component of disorder. ** average distances between nitrogen and carbon atoms (N-Cendo—between secondary amine nitrogen atoms and skeleton carbon atoms; NBz-C—between benzyl substituted nitrogen atoms and skeleton carbon atoms; N-CO—between amide nitrogen atoms and carbonyl carbon atoms). *** the sum of C-N-C angles (NBz—benzyl substituted nitrogen atom; Namid—amide nitrogen atom; NH—secondary amine nitrogen atom). **** length of hydrogen bonds. ***** intramolecular distance between nitrogen atoms.

## Supplementary Materials

The following are available online at http://www.mdpi.com/2079-4991/9/1/89/s1. Figure S1: Photo of **4ae*HCl** in nitrobenzene, Figure S2: Photo of **4ae*HCl** in ethoxybenzene, Figure S3: Photo of **4ae*HCl** in mesitylene, Figure S4: Photo of **4ab*HCl** in nitrobenzene, Figure S5: Photo of **4ab*HCl** in ethoxybenzene, Figure S6: Photo of **4ab*HCl** in mesitylene, Figure S7: POM photomicrography, Figure S8: Dependence of viscosity on shear rate, Figure S9: Dependence of the loss and accumulation modules on angular frequency, Figure S10: Molecular structure of **4cb**, Figure S11: Molecular structure of **2c**, Figure S12: Molecular structure of **3a**, Figure S13: Molecular structure of **3c**, Figure S14: Molecular structure of **4da*HCl*(C_6_H_6_)_2_**, Figure S15: Hydrogen-bonded finite motif in the structure **1c+H2O**, Figure S16: Molecular structure of 5,7-dimethyl-1,3-diazaadamantan-6-one, Figure S17: The structures a) and b) of ketone and its diol form in the crystal 1c, Figure S18: Scatterplot of C9…N separations in structures of neutral organic flexible bispidines, Figure S19: Histogram of absolute differences between C9…N separations, Figure S20: TEM micrograph of **4bc*HCl**, Figure S21–S23: AFM micrograph of **4bc*HCl**, Figure S24: SEM micrographs of dry gel samples obtained by different solvent removal methods from **ethoxybenzene@4ae*HCl**, Figure S25: FTIR spectra of the native gels **benzene@4de*HCl** and **benzene@4ce*HCl**, Table S1: Dependence of shear rate on viscosity, Table S2: Dependence of the loss and storage moduli on angular frequency, Table S3: Crystal data, data collection, structure solution, and refinement parameters for **4cb**, **2c**, **3a**, **3c**, **1b**, **5,7-dimethyl-1,3-diazaadamantan-6-one**, and **4da**.

## References

[1] Hendel R.C., Kimmelstiel C. (2017). Cardiology Procedures.

[2] Vatsadze S.Z., Eremina O.E., Veselova I.A., Kalmykov S.N., Nenajdenko V.G. (2018). ^18^F-Labelled catecholamine type radiopharmaceuticals in the diagnosis of neurodegenerative diseases and neuroendocrine tumours: Approaches to synthesis and development prospects. Russ. Chem. Rev..

[3] Jødal L., Le Loirec C., Champion C. (2014). Positron range in PET imaging: Non-conventional isotopes. Phys. Med. Biol..

[4] Holland J.P., Williamson M.J., Lewis J.S. (2010). Unconventional Nuclides for Radiopharmaceuticals. Mol. Imaging.

[5] Schubiger A.P., Lehmann L., Friebe M. (2007). PET Chemistry (Ernst Schering Research Foundation Workshop 62).

[6] Paterson B.M., Donnelly P.S. (2016). Macrocyclic Bifunctional Chelators and Conjugation Strategies for Copper-64 Radiopharmaceuticals. Advances in Inorganic Chemistry.

[7] Lee S.G., Gangangari K., Kalidindi T.M., Punzalan B., Larson S.M., Pillarsetty N.V.K. (2016). Copper-64 labeled liposomes for imaging bone marrow. Nucl. Med. Biol..

[8] Comba P., Kerscher M., Rück K., Starke M. (2018). Bispidines for radiopharmaceuticals. Dalton Trans..

[9] Comba P., Jermilova U., Orvig C., Patrick B.O., Ramogida C.F., Rück K., Schneider C., Starke M. (2017). Octadentate Picolinic Acid-Based Bispidine Ligand for Radiometal Ions. Chem. A Eur. J..

[10] Medved’Ko A.V., Egorova B.V., Komarova A.A., Rakhimov R.D., Krut’Ko D.P., Kalmykov S.N., Vatsadze S.Z. (2016). Copper-Bispidine Complexes: Synthesis and Complex Stability Study. ACS Omega.

[11] Stephan H., Walther M., Fahnemann S., Ceroni P., Molloy J.K., Bergamini G., Heisig F., Muller C.E., Kraus W., Comba P. (2014). Bispidines for Dual Imaging. Chem. A Eur. J..

[12] Tomassoli I., Gündisch D. (2016). Bispidine as a Privileged Scaffold. Curr. Top. Med. Chem..

[13] Comba P., Morgen M., Wadepohl H. (2013). Tuning of the properties of transition-metal bispidine complexes by variation of the basicity of the aromatic donor groups. Inorg. Chem..

[14] Toom L., Kütt A., Kaljurand I., Leito I., Ottosson H., Grennberg H., Gogoll A. (2006). Substituent Effects on the Basicity of 3,7-Diazabicyclo[3.3.1]nonanes. J. Org. Chem..

[15] Breuning M., Steiner M. (2008). Chiral Bispidines. Synthesis.

[16] Liu J., Yang Z., Wang Z., Wang F., Chen X., Liu X., Feng X., Su Z., Hu C. (2008). Asymmetric Aldol Reaction Using New Bispidine Catalysts. Synfacts.

[17] Haridas V., Sadanandan S., Sharma Y.K., Chinthalapalli S., Shandilya A. (2012). Bispidine as a secondary structure nucleator in peptides. Tetrahedron Lett..

[18] Zhang Y.C., Gao J.Y., Shi N.Y., Zhao J.Q. (2011). Synthesis of Chiral Tridentate Ligands Embodying the Bispidine Framework and their Application in the Enantioselective Addition of Diethylzinc to Aldehydes. Adv. Mater. Res..

[19] Stucchi M., Lesma G. (2016). Split- Ugi Reaction with Chiral Compounds: Synthesis of Piperazine- and Bispidine-Based Peptidomimetics. Helv. Chim. Acta.

[20] Rossetti A., Landoni S., Meneghetti F., Castellano C., Mori M., Colombo Dugoni G., Sacchetti A. (2018). Application of chiral bi- and tetra-dentate bispidine-derived ligands in the copper(II)-catalyzed asymmetric Henry reaction. New J. Chem..

[21] Zefirov N.S., Palyulin V.A. (1991). Topics in Stereochemistry. Top. Stereochem..

[22] Vatsadze S.Z., Krut’ko D.P., Zyk N.V., Zefirov N.S., Churakov A.V., Howard J.A. (1999). First 1H NMR observation of chair-boat conformers in bispidinone system. Molecular structure of 3,7-diisopropyl-1,5-diphenyl-3,7-diazabicyclo-[3.3.1]nonane-9-one. Mendeleev Commun..

[23] Palyulin V.A., Emets S.V., Chertkov V.A., Kasper C., Schneider H.J. (1999). Conformational switching of 3,7-diacyl-3,7-diazabicyclo[3.3.1]nonanes by metal binding and by solvent changes. Eur. J. Org. Chem..

[24] Kudryavtsev K.V., Shulga D.A., Chupakhin V.I., Sinauridze E.I., Ataullakhanov F.I., Vatsadze S.Z. (2014). Synthesis of novel bridged dinitrogen heterocycles and their evaluation as potential fragments for the design of biologically active compounds. Tetrahedron.

[25] Vatsadze S.Z., Shulga D.A., Loginova Y.D., Vatsadze I.A., Wang L., Yu H., Kudryavtsev K.V. (2016). Computer modeling of ferrocene-substituted 3,7-diazabicyclo[3.3.1]nonanes as serine protease inhibitors. Mendeleev Commun..

[26] Comba P., Kerscher M., Schiek W. (2007). Bispidine Coordination Chemistry. Prog. Inorg. Chem..

[27] Comba P., Jakob M., Rück K., Wadepohl H. (2017). Tuning of the properties of a picolinic acid-based bispidine ligand for stable copper(II) complexation. Inorg. Chim. Acta.

[28] Comba P., Hunoldt S., Morgen M., Pietzsch J., Stephan H., Wadepohl H. (2013). Optimization of Pentadentate Bispidines as Bifunctional Chelators for 64 Cu Positron Emission Tomography (PET). Inorg. Chem..

[29] Atanasov M., Comba P., Martin B., Müller V., Rajaraman G., Rohwer H., Wunderlich S. (2006). DFT models for copper(II) bispidine complexes: Structures, stabilities, isomerism, spin distribution, and spectroscopy. J. Comput. Chem..

[30] Amabilino D.B., Smith D.K., Steed J.W. (2017). Supramolecular materials. Chem. Soc. Rev..

[31] Vieira V.M.P., Hay L.L., Smith D.K. (2017). Multi-component hybrid hydrogels – understanding the extent of orthogonal assembly and its impact on controlled release. Chem. Sci..

[32] Hirst A.R., Escuder B., Miravet J.F., Smith D.K. (2008). High-tech applications of self-assembling supramolecular nanostructured gel-phase materials: From regenerative medicine to electronic devices. Angew. Chem. (Int. Ed. Engl.).

[33] Draper E.R., Adams D.J. (2017). Low-Molecular-Weight Gels: The State of the Art. Chem.

[34] Weiss R.G. (2014). The past, present, and future of molecular gels. What is the status of the field, and where is it going?. J. Am. Chem. Soc..

[35] Sangeetha N.M., Maitra U. (2005). Supramolecular gels: Functions and uses. Chem. Soc. Rev..

[36] Abdallah D.J., Weiss R.G. (2000). Organogels and Low Molecular Mass Organic Gelators. Adv. Mater..

[37] Terech P., Weiss R.G. (1997). Low Molecular Mass Gelators of Organic Liquids and the Properties of Their Gels. Chem. Rev..

[38] Smith D.K., Atwood J.L., Steed J.W. (2008). Molecular Gels—Nanostructured Soft Materials. Organic Nanostructures.

[39] Cornwell D.J., Smith D.K. (2015). Expanding the scope of gels – combining polymers with low-molecular-weight gelators to yield modified self-assembling smart materials with high-tech applications. Mater. Horiz..

[40] Hirst A.R., Coates I.A., Boucheteau T.R., Miravet J.F., Escuder B., Castelletto V., Hamley I.W., Smith D.K. (2008). Low-molecular-weight gelators: Elucidating the principles of gelation based on gelator solubility and a cooperative self-assembly model. J. Am. Chem. Soc..

[41] Ananikov V.P., Khemchyan L.L., Ivanova Y.V., Bukhtiyarov V.I., Sorokin A.M., Prosvirin I.P., Vatsadze S.Z., Medved’ko A.V., Nuriev V.N., Dilman A.D. (2014). Development of new methods in modern selective organic synthesis: Preparation of functionalized molecules with atomic precision. Russ. Chem. Rev..

[42] Desvergne J.-P. (2010). Organic gelators and hydrogelators. Beilstein J. Org. Chem..

[43] Smith D.K. (2007). Molecular gels-underpinning nanoscale materials with organic chemistry. Tetrahedron.

[44] Liu X.Y. (2005). Low Molecular Mass Gelator.

[45] Weiss R.G., Terech P. (2006). Molecular Gels: Materials with Self-Assembled Fibrillar Networks.

[46] Jones C.D., Steed J.W. (2016). Gels with sense: Supramolecular materials that respond to heat, light and sound. Chem. Soc. Rev..

[47] Yang H., Yi T., Zhou Z., Zhou Y., Wu J., Xu M., Li F., Huang C. (2007). Switchable fluorescent organogels and mesomorphic superstructure based on naphthalene derivatives. Langmuir ACS J. Surf. Colloids.

[48] Sahoo P., Sankolli R., Lee H.-Y., Raghavan S.R., Dastidar P. (2012). Gel sculpture: Moldable, load-bearing and self-healing non-polymeric supramolecular gel derived from a simple organic salt. Chemistry.

[49] Cornwell D.J., Okesola B.O., Smith D.K. (2014). Multidomain hybrid hydrogels: Spatially resolved photopatterned synthetic nanomaterials combining polymer and low-molecular-weight gelators. Angew. Chem. Int. Ed..

[50] Morris K.L., Chen L., Raeburn J., Sellick O.R., Cotanda P., Paul A., Griffiths P.C., King S.M., O’Reilly R.K., Serpell L.C. (2013). Chemically programmed self-sorting of gelator networks. Nat. Commun..

[51] Lee J.H., Jung S.H., Lee S.S., Kwon K.-Y., Sakurai K., Jaworski J., Jung J.H. (2017). Ultraviolet Patterned Calixarene-Derived Supramolecular Gels and Films with Spatially Resolved Mechanical and Fluorescent Properties. Acs Nano.

[52] Ajayaghosh A., Praveen V.K., Vijayakumar C. (2008). Organogels as scaffolds for excitation energy transfer and light harvesting. Chem. Soc. Rev..

[53] Okesola B.O., Smith D.K. (2016). Applying low-molecular weight supramolecular gelators in an environmental setting—self-assembled gels as smart materials for pollutant removal. Chem. Soc. Rev..

[54] Vieira V., Lima A., de Jong M., Smith D.K. (2018). Commercially-relevant orthogonal multi-component supramolecular hydrogels for programmed cell growth. Chem. A Eur. J..

[55] Vatsadze S.Z., Tyurin V.S., Zatsman A.I., Manaenkova M.A., Semashko V.S., Krut’ko D.P., Zyk N.V., Churakov A.V., Kuz’mina L.G. (2006). New stereoselective intramolecular redox reaction in the system of 3,7-diazabicyclo[3.3.1]nonan-9-one. Russ. J. Org. Chem..

[56] Semashko V.S., Vatsadze S.Z., Zyk N.V., Godovikov I.A. (2008). An NMR study of quaternary ammonium salts of 5,7-dimethyl-1,3- diazaadamantan-6-one. Russ. Chem. Bull..

[57] Balakhonov S.V., Vatsadze S.Z., Churagulov B.R. (2015). Effect of supercritical drying parameters on the phase composition and morphology of aerogels based on vanadium oxide. Russ. J. Inorg. Chem..

[58] Shlyakhtin A.V., Vatsadze S.Z., Krut’ko D.P., Lemenovskii D.A., Zabalov M.V. (2012). Carboxylation of aromatic compounds in a supercritical carbon dioxide medium. Russ. J. Phys. Chem. B.

[59] Sheldrick G.M. (2008). A short history of SHELX. Acta Crystallogr. Sect. A.

[60] Yu G., Yan X., Han C., Huang F. (2013). Characterization of supramolecular gels. Chem. Soc. Rev..

[61] Draper E.R., Adams D.J. (2018). How should multicomponent supramolecular gels be characterised?. Chem. Soc. Rev..

[62] Dawn A., Kumari H. (2018). Low Molecular Weight Supramolecular Gels Under Shear: Rheology as the Tool for Elucidating Structure–Function Correlation. Chem. A Eur. J..

[63] Cui H., Goddard R., Pörschke K.R. (2012). Degradation of dichloromethane by bispidine. J. Phys. Org. Chem..

[64] Zefirov N.S., Palyulin V.A., Iliel E.L., Wilen S.H. (1991). Conformational Analysis of Bicyclo[3.3.1]nonanes and Their Hetero Analogs. Topics in Stereochemistry.

[65] Groom C.R., Bruno I.J., Lightfoot M.P., Ward S.C. (2016). The Cambridge structural database. Acta Crystallogr. Sect. B: Struct. Sci. Cryst. Eng. Mater..

[66] Kudryavtsev K.V., Vatsadze S.Z., Semashko V.S., Churakov A.V. (2012). *syn* -3-(4-Chlorobenzyl)-1,5-dimethyl-3,7-diazabicyclo[3.3.1]nonan-9-ol. Acta Crystallogr. Sect. E Struct. Rep. Online.

[67] Maekawa H., Nishiyama Y. (2015). Selective introduction of a trifluoroacetyl group onto 4-vinylpyridines through magnesium-promoted reduction. Tetrahedron.

[68] Ebead A., Fournier R., Lee-Ruff E. (2011). Synthesis of cyclobutane nucleosides. Nucleosidesnucleotides Nucl. Acids.

[69] Kurkutova E.N., Goncharov A.V., Zefirov N.S., Palyulin V.A. (1976). Molecular Structure of 1-Methyl-5,7- diphenyl-1,3-diazaadamantan-6-one Iodide. J. Struct. Chem..

