# Supplementary material for: Supramolecular Organogels Based on N-Benzyl, N′-Acylbispidinols
Source: Nanomaterials (Basel). 2019 Jan 11;9(1):89. doi: 10.3390/nano9010089 (PMC6359647; doi:10.3390/nano9010089)
Supplement: Supplementary file 1 [file nanomaterials-09-00089-s001.pdf]

Supplementary Information

Supramolecular Organogels Based on *N*-Benzyl, *N′*-Acylbispidinols

Alexey V. Medved′ko [1], Alexander I. Dalinger [1], Vyacheslav N. Nuriev [1], Vera S. Semashko [1], Andrei V. Filatov [2], Alexander A. Ezhov [3,4], Andrei V. Churakov [5], Judith A. K. Howard [6], Andrey A. Shiryaev [7,8], Alexander E. Baranchikov [1,5], Vladimir K. Ivanov [5,9], and Sergey Z. Vatsadze [1,*]

1 Faculty of Chemistry, Lomonosov Moscow State University, 119991 Moscow, Russia; lexeym@gmail.com (A.V.M.); dal1995@mail.ru (A.I.D.); nvn@org.chem.msu.ru (V.N.N.); vera-s@yandex.ru (V.S.S.); a.baranchikov@yandex.ru (A.E.B.)
2 Zelinsky Institute of Organic Chemistry, Russian Academy of Sciences, 119991 Moscow, Russia; filatov_andrey_@mail.ru
3 Faculty of Physics, Lomonosov Moscow State University, 119991 Moscow, Russia; alexander-ezhov@yandex.ru
4 Topchiev Institute of Petrochemical Synthesis, Russian Academy of Sciences, 119991 Moscow, Russia
5 Kurnakov Institute of General and Inorganic Chemistry of the Russian Academy of Sciences, 119991 Moscow, Russia; churakov@igic.ras.ru (A.V.C.); van@igic.ras.ru (V.K.I.)
6 Department of Chemistry, University of Durham, Durham DH1 3LE, UK; j.a.k.howard@durham.ac.uk
7 Frumkin Institute of Physical Chemistry and Electrochemistry, Russian Academy of Sciences, 119071 Moscow, Russia; a_shiryaev@mail.ru
8 Institute of Geology of Ore Deposits, Petrography, Mineralogy and Geochemistry, Russian Academy of Sciences, 119017 Moscow, Russia
9 Faculty of Material Science, Lomonosov Moscow State University, 119991 Moscow, Russia
* Correspondence: szv@org.chem.msu.ru; Tel.: +7-903-748-7892

**Preparation of unsymmetrical bispidines**	**pp. 3–10**
**Figure S1–S2**	**p. 7**
**Figure S3**	**p. 8**
**Figure S4–S5**	**p. 9**
**Figure S6**	**p. 10**
**Figure S7**	**p. 11**
**Table S1**	**p. 12**
**Figure S8**	**p. 12**
**Table S2**	**p. 13**
**Figure S9**	**p. 13**
**Table S3**	**pp. 14–15**
**Figure S10–S12**	**p. 16**
**Figure S13–S15**	**p. 17**
**Figure S16–S17**	**p. 18**
**Figure S18–S19**	**p. 19**
**Figure S20–S21**	**p. 20**
**Figure S22–S24**	**p. 21**
**Figure S25**	**p. 22**

Preparation of unsymmetrical bispidines. General method.

To a suspension of bispidine **3** in dry benzene was added a solution of acyl halide (1 eq) in dry benzene. Then the mixture was refluxed under vigorous stirring for 3.5 hours. To obtain the product as the hydrochloride, the resulting gelatinous mass (precipitate or colloidal solution) was centrifuged for 10-15 minutes (6000 rpm) or filtered on a Schott filter (por.40) and then was dried. The free base was isolated from the aqueous solution by treatment with sodium bicarbonate (3 eq.).

Anti-1,5-dimethyl-3-benzoyl-7-benzyl-3,7-diazabicyclo[3.3.1]nonane-9-ol (4aa).

140 mg (0.54 mmol) of bispidine **3a** and 75.6 mg (0.54 mmol) of benzoyl chloride in 10 ml of dry benzene give colloidal solution, from which 0.134 g of the free base was isolated. Yield 68%.

NMR ^{1}H (DMSO-d_6, δ/ ppm, *J*/Hz): 0.65 (s, 3H, CH_3); 0.79 (s, 3H, CH_3); 2.13 (d, 1H, H6e/8e/6a/8a, *J* = 10.2 Hz); 2.24-2.32 (m, 2H, H6e/8e/6a/8a); 2.37 (d, 1H, H6e/8e/6a/8a, *J* = 11.3 Hz); 2.65 (d, 1H, H4a, *J* = 12,5 Hz); 3.04 (d, 1H, H2a, *J* = 12.5 Hz); 3.13-3.18 (m, 2H, CH_2Ph, H9); 3.42 (d, 1H, H2e, *J* = 12.9 Hz); 3.54 (d, 1H, CH_2Ph, *J* = 13.3 Hz); 4.47 (d, 1H, H4e, *J* = 13.2 Hz); 4,95 (d, 1H, OH, *J* = 4.7 Hz), 7.21-7.39 (m, 10H, H(Ar)).

NMR ^{13}C (characteristic signals) (DMSO-d_6, δ/ ppm, *J*/Hz): 21.5 (s, CH_3); 21.8 (s, CH_3); 35.17 (br. s, C1, C5); 52.2 (s, C2); 57.3 (s, C4); 57.8 (s, C6); 58.1 (s, C8); 63.4 (s, CH_2Ph); 75.5 (s, C9); 126.2 (s, Ar), 128.3 (s, Ar), 129.0 (s, Ar), 130.16 (s, Ar), 130.45 (s, Ar), 130,56 (s, Ar), 133.15 (s, Ar); 168,5 (s, C=O).

Found (%): C, 75.80; H, 7.95; N, 7.46. Calculated for $C_{23}H_{28}N_2O_2$ (%): C, 75.79; H, 7.74; N, 7.69.

Anti-1,5-dimethyl-3-benzoyl-7-(4-fluorobenzyl)-3,7-diazabicyclo[3.3.1]nonane-9-ol (4ca)

70 mg (0.25 mmol) of bispidine **3c** and 35.4 mg (0.25 mmol) of benzoyl chloride in 5 ml of dry benzene give colloidal solution, from which 0.082 g of the free base was isolated. Yield 85%.

NMR ^{1}H (DMSO-d_6, δ/ ppm, *J*/Hz): 0.65 (s, 3H, CH3), 0.79 (s, 3H, CH_3); 2.13 (d, 1H, H6e/8e/6a/8a, *J* = 10.2 Hz); 2.24-2.32 (m, 3H, H6e/8e/6a/8a); 2.65 (d, 1H, H4a, *J* = 12.9 Hz); 3.05 (d, 1H, H2a, *J* = 12.9 Hz); 3.13-3.17 (m, 2H, CH_2Ph, H9); 3.42 (d, 1H, H2e, *J* = 13.3 Hz); 3.52 (d, 1H, CH_2Ph, *J* = 12.9 Hz); 4.48 (d, 1H, H4e, *J* = 13.1 Hz); 4,95 (d, 1H, OH, *J* = 4.5 Hz); 7.08 (tr, 2H, CH(3,5) (C_6H_4F) *J* = 8,4 Hz); 7.29-7.41 (m, 6H, H(Ar)).

NMR ^{13}C (characteristic signals) (DMSO-d_6, δ/ ppm, *J*/Hz): 21.4, 21.8 (both s, $2CH_3$); 36.1, 36.6 (both s, C1, C5); 52.1, 57.1, 57.8, 58.1 (all s, C2, C4, C6, C8); 62.5 (s, CH_2Ph); 75.95 (s, C9); 127, 128.7, 129, 132.3, 130.85, 130.93, 134.9, 137.74 (all s, Ar); 168,5 (s, C=O).

Found (%):C, 72.57; H, 7.09; N, 7.12. Calculated for $C_{23}H_{27}FN_2O_2$ (%):C, 72.23; H, 7.12; N, 7.32.

Anti-1,5-dimethyl-3-benzoyl-7-(4-chlorobenzyl)-3,7-diazabicyclo[3.3.1]nonane-9-ol (4ba)

70 mg (0.24 mmol) of bispidine **3b** and 33.4 mg (0.24 mmol) of benzoyl chloride in 5 ml of dry benzene give colloidal solution, from which 0.084 g of the free base was isolated. Yield 88%.

NMR ^{1}H (DMSO-d_6, δ/ ppm, *J*/Hz): 0.65, 0.79 (both s, 6H, $2CH_3$); 2.13 (d, 1H, H6e/8e/6a/8a, *J* = 10.8 Hz); 2.23-2.36 (m, 3H, H6e/8e/6a/8a); 2.65 (d, 1H, H4a, *J* = 12.5 Hz); 3.05 (d, 1H, H2a, *J* = 13.9 Hz); 3.13-3.17 (m, 2H, CH_2Ph, H9); 3.44 (d, 1H, H2e, *J* = 14.0 Hz); 3.53 (d, 1H, CH_2Ph, *J* = 13.9 Hz); 4.49 (d, 1H, H4e, *J* = 12.9 Hz); 4,95 (d, 1H, OH, *J* = 5.28 Hz); 7.29-7.41 (m, 9H, CH(Ar)).

NMR ^{13}C (characteristic signals) (DMSO-d_6, δ/ ppm, *J*/Hz): 21.4, 21.8 (both s, $2CH_3$); 36.1, 36.6 (both s, C1, C5); 54.9, 55.7, 57.1, 58.1 (all s, C2, C4, C6, C8); 62.5 (s, CH_2Ph); 75.9 (s, C9); 127, 128.5, 128.74, 129.25, 132.3, 130.85, 130.93, 137.71(br.) (all s, Ar); 168,5 (s, C=O).

Found (%): C, 69.35; H, 6.95; N, 6.93. Calculated for $C_{23}H_{27}ClN_2O_2$ (%): C, 69.25; H, 6.82; N, 7.02.

Anti-1,5-dimethyl-3-benzoyl-7-(4-bromobenzyl)-3,7-diazabicyclo[3.3.1]nonane-9-ol (4da)

140 mg (0.4 mmol) of bispidine **3d** and 56.5 mg (0.4 mmol) of benzoyl chloride in 10 ml of dry benzene give gel, from which 0.14 g of the free base was isolated. Yield 79%.

NMR ^1H (DMSO-d_6, δ/ ppm, *J*/Hz): 0.65, 0.79 (both s, 6H, $2CH_3$); 2.15 (d, 1H, H6e/8e/6a/8a, *J* = 10.2 Hz); 2.23-2.36 (m, 3H, H6e/8e/6a/8a); 2.65 (d, 1H, H4a, *J* = 12.5 Hz); 3.05 (d, 1H, H2a, *J* = 13.3 Hz); 3.12-3.15 (m, 2H, CH_2Ph, H9); 3.42 (d, 1H, H2e, *J* = 13.7 Hz); 3.53 (d, 1H, CH_2Ph, *J* = 13.9 Hz); 4.48 (d 1H, H4e, *J* = 12.1 Hz); 4,95 (d, 1H, OH, *J* = 5.09 Hz); 7.29-7.31 (m, 4H, CH(Ar)); 7.40-7.41 (m, 3H, CH(Ar)); 7.44-7.46 (d, 2 H, CH(2,6)(Ar), *J* = 8.4 Hz).

NMR ^{13}C (characteristic signals) (DMSO-d_6, δ/ ppm, *J*/Hz): 21.4, 21.8 (both s, $2CH_3$); 36.1, 36.6 (both s, C1, C5); 54.6, 57.1, 58.1 (all s, C2, C4, C6, C8); 75.90 (s, C9); 127, 128.7, 131.3, 131.41 (all s, Ar).

Found (%): C, 62.32; H, 6.14; N, 6.10. Calculated for $C_{23}H_{27}BrN_2O_2$ (%): C, 62.31; H, 6.14; N, 6.32.

Anti-1,5-dimethyl-3-(4-chlorobenzoyl)-7-benzyl-3,7-diazabicyclo[3.3.1]nonane-9-ol (4ab)

70 mg (0.27 mmol) of bispidine **3a** and 47.1 mg (0.27 mmol) of 4-chlorobenzoyl chloride in 5 ml of dry benzene give gel, from which 0.093 g of the free base was isolated. Yield 78%.

NMR ^1H (DMSO-d_6, δ/ ppm, *J*/Hz): 0.66, 0.79 (both s, 6H, $2CH_3$); 2.13 (d, 1H, H6e/8e/6a/8a, *J* = 10.9 Hz); 2.25-2.32 (m, 2H, H6e/8e/6a/8a); 2.37 (d, 1H, H6e/8e/6a/8a, *J* = 10.6 Hz); 2.65 (d, 1H, H4a, *J* = 12.9 Hz); 3.06 (d, 1H, H2a, *J* = 13.3 Hz); 3.12-3.18 (m, 2H, CH_2Ph, H9); 3.37 (d, 1H, H2e, *J* = 13.5 Hz); 3.52 (d, 1H, CH_2Ph, *J* = 13.3 Hz); 4.45 (d, 1H, H4e, *J* = 12.9 Hz); 4,95 (d, 1H, OH, *J* = 4.9 Hz); 7.29-7.31 (m, 4H, CH(Ar)); 7.22-7.34 (m, 7H, CH(Ar)); 7.44-7.46 (d, 2 H, CH(2,6)(Ar), *J* = 8.2 Hz).

NMR ^{13}C (characteristic signals) (DMSO-d_6, δ/ ppm, *J*/Hz): 21.4, 21.8 (both s, $2CH_3$); 36.1, 36.6 (s, C1, C5); 52.2, 57.3, 57.8, 58.1 (all s, C2, C4, C6, C8); 63.4 (s, CH_2Ph); 75.9 (s, C9); 127.2, 128.6, 128.9, 129.0, 133.8, 136.5, 138,77 (all s, Ar); 167,5 (s, C=O).

Found (%): C, 69.48; H, 6.93; N, 6.93. Calculated for $C_{23}H_{27}ClN_2O_2$ (%): C, 69.25; H, 6.82; N, 7.02.

Anti-1,5-dimethyl-3-(4-chlorobenzoyl)-7-(4-fluorobenzyl)-3,7-diazabicyclo[3.3.1]nonane-9-ol (4cb)

70 mg (0.25 mmol) of bispidine **3c** and 44.1 mg (0.25 mmol) of 4-chlorobenzoyl chloride in 5 ml of dry benzene give gel, from which 0.089 g of the free base was isolated. Yield 92%.

NMR ^1H (DMSO-d_6, δ/ ppm, *J*/Hz): 0.66, 0.78 (both, 6H, 2CH3); 2.13 (d, 1H, H6e/8e/6a/8a, *J* = 11.49 Hz); 2.23-2.36 (m, 3H, H6e/8e/6a/8a); 2.68 (d, 1H, H4a, *J* = 13.45 Hz); 3.06-3.15 (m, 3H, H2a, CH2Ph, H9); 3.41 (d, 1H, H2e, *J* = 13.33 Hz); 3.54 (d, 1H, CH_2Ph, *J* = 13.57 Hz); 4.48 (d, 1H, H4e, *J* = 14.18 Hz); 4,98 (d, 1H, OH, *J* = 5.14 Hz); 7.09 (tr, 2H, CH(3,5) (C_6H_4F) *J* = 8,86 Hz); 7.31-7.37 (m, 4H, H(Ar)); 7.49 (d, 2H, H(Ar), *J*=8.44 Hz).

NMR ^{13}C (characteristic signals) (DMSO-d_6, δ/ ppm, *J*/Hz): 21.4, 21.8 (both s, $2CH_3$); 36.1, 36.6 (s, C1, C5); 52.2, 57.0, 57.8, 58.0 (all s, C2, C4, C6, C8); 62.5 (s, CH_2Ph); 75.9 (s, C9); 128.9, 129.0, 133.9, 136.5 (all s, Ar); 167,5 (s, C=O).

Found (%): C, 66.37; H, 6.35; N, 6.61. Calculated for $C_{23}H_{26}ClFN_2O_2$ (%): C, 66.26; H, 6.29; N, 6.72.

Anti-1,5-dimethyl-3-(4-chlorobenzoyl)-7-(4-chlorobenzyl)-3,7-diazabicyclo[3.3.1]nonane-9-ol (4bb)

70 mg (0.24 mmol) of bispidine **3b** and 41.7 mg (0.24 mmol) of 4-chlorobenzoyl chloride in 5 ml of dry benzene give gel, from which 0.072 g of the free base was isolated. Yield 69%.

NMR ^1H (DMSO-d_6, δ/ ppm, *J*/Hz): 0.66, 0.79 (both s, 6H, $2CH_3$); 2.14 (d, 1H, H6e/8e/6a/8a, *J* = 11.49 Hz); 2.24-2.35 (m, 3H, H6e/8e/6a/8a); 2.68 (d, 1H, H4a, *J* = 13.45 Hz); 3.09 (д, 1H, H2a, *J* = 13.45 Hz); 3.12-3.16 (m, 2H, CH_2Ph, H9); 3.41 (d, 1H, H2e, *J* = 12.69 Hz); 3.55 (d, 1H, CH_2Ph, *J* = 13.45 Hz);

4.48 (d, 1H, H4e, *J* = 13.45 Hz); 4,98 (d, 1H, OH, *J* = 5.01 Hz); 7.31-7.37 (m, 6H, CH(Ar)), 7.47(d, 2H, H(2,6)(ArC=O), *J* = 8.19 Hz).

NMR ^{13}C (characteristic signals) (DMSO-d_6, δ/ ppm, *J*/Hz): 21.4, 21.7 (both s, $2CH_3$); 36.6 (br. s, C1, C5); 52.1, 53.9, 57.1, 58.0 (all s, C2, C4, C6, C8); 62.45 (s, CH_2Ph); 75.8 (s, C9); 128.5, 128.9, 130.9, 137.8 (all s, Ar); 167,5 (s, C=O).

Found (%): C, 63.70; H, 6.25; N, 6.34. Calculated for $C_{23}H_{26}Cl_2N_2O_2$ (%): C, 63.74; H, 6.05; N, 6.46.

Anti-1,5-dimethyl-3-(4-chlorobenzoyl)-7-(4-bromobenzyl)-3,7-diazabicyclo[3.3.1]nonane-9-ol (4db)

140 mg (0.4 mmol) of bispidine **3d** and 72.2 mg (0.4 mmol) of 4-chlorobenzoyl chloride in 5 ml of dry benzene give gel, from which 0.149 g of the free base was isolated. Yield 79%.

NMR ^1H (DMSO-d_6, δ/ ppm, *J*/Hz): 0.66, 0.79 (both s, 6H, 2CH3); 2.12 (d, 1H, 6a/6e/8a/8e, *J* = 9.78 Hz); 2.23-2.35 (m, 3H, 6a/6e/8a/8e); 2.65 (d, 1H, H4a, *J* = 13.30 Hz); 3.06-3.15 (m, 3H, H2a, H9, -CH_2-Ar); 3.38 (d, 1H, ,H2e, *J* = 13.5 Hz); 3.49 (d, 1H, -CH_2-Ar, *J* = 13.30 Hz); 4.45 (d, 1H, 4He, *J* = 13,69 Hz); 4.96 (d, 1H, -OH, *J* = 5.09 Hz); 7.26-7.32 (m, 4H, Ar-Br); 7.45-7.48 (m, 4H, Ar-Cl).

NMR ^{13}C (characteristic signals) (DMSO-d_6, δ/ ppm, *J*/Hz): 21.2, 21.7 (both s, $2CH_3$); 36.1 (br. s, C1, C5); 128.9, 129.0, 131.4, 131.6 (all s, Ar); 171,9 (s, C=O).

Found (%): C, 57.69; H, 5.51; N, 5.85. Calculated for $C_{23}H_{26}BrClN_2O_2$ (%): C, 57.81; H, 5.48; N, 5.86.

Anti-1,5-dimethyl-3-(2-thiophenecarbonyl)-7-benzyl-3,7-diazabicyclo[3.3.1]nonane-9-ol (4ae)

70 mg (0.27 mmol) of bispidine **3a** and 39.3 mg (0.27 mmol) of 2-thiophenecarbonyl chloride in 5 ml of dry benzene give gel, from which 0.081 g of the free base was isolated. Yield 81%.

NMR ^1H (DMSO-d_6, δ/ ppm, *J*/Hz): 0.72, 0.80 (both s, 6H, $2CH_3$); 2.16 (d, 1H, H6e/8e/6a/8a, *J* = 13.69 Hz); 2.24-2.30 (m, 2H, H6e/8e/6a/8a); 2.35 (d, 2H, H6e/8e/6a/8a, *J* = 12.32 Hz); 2.67 (d, 1H, H4a, *J* = 13.3 Hz); 3.16 (d, 1H, H9, *J* = 5.09 Hz); 3.19-3.24 (m, 2H, H2a, CH_2Ph); 3.37 (H2e, under H_2O signal); 3.90 (d, 1H, CH_2Ph, *J* = 13.3 Hz); 4.49 (d, 1H, H4e, *J* = 12.9 Hz); 4,96 (d, 1H, OH, *J* = 5.28 Hz); 7.07-7.09 (dd, 1H, CH(4)(C_4H_3S), J_1 = 5.0 Hz, J_2 = 3.81 Hz), 7.16-7.28 (m, 6H, CH(Ph), CH(3)(C_4H_3S)), 7.70 (d, 1H, CH(5)(C_4H_3S), *J*=4.9 Hz).

NMR ^{13}C (characteristic signals) (DMSO-d_6, δ/ ppm, *J*/Hz): 21.5, 21.7 (both s, $2CH_3$); 36.2, 36.7 (s, C1, C5); 52.8, 57.6, 57.9 (br.) (all s, C2, C4, C6, C8); 63.4 (s, CH_2Ph); 75.9 (s, C9); 127.0, 127.2, 128.5, 128.7, 128.9, 129.0, 138.9 (all s, Ar); 162,2 (s, C=O)

Found (%): C, 68.43; H, 7.10; N, 7.35; S, 8.60. Calculated for $C_{21}H_{26}N_2O_2S$ (%): C, 68.08; H, 7.07; N, 7.56; S, 8.65.

Anti-1,5-dimethyl-3-(2-thiophenecarbonyl)-7-(4-fluorobenzyl)-3,7-diazabicyclo[3.3.1]nonane-9-ol (4ce)

70 mg (0.25 mmol) of bispidine **3c** and 36.8 mg (0.25 mmol) of 2-thiophenecarbonyl chloride in 5 ml of dry benzene give gel, from which 0.085 g of the free base was isolated. Yield 88%.

NMR ^1H (DMSO-d_6, δ/ ppm, *J*/Hz): 1.49, 1.56 (both s, 6H, 2CH3); 2.93 (d, 1H, H6e/8e/6a/8a, *J* = 8.19 Hz); 3.02 (m, 2H, H6e/8e/6a/8a), 3.12 (d, 1H, H6e/8e/6a/8a, *J* = 10.76 Hz), 3.43 (d, 1H, H4a, *J* = 12.84 Hz); 3.93-4.01 (m, 3H, H2a, CH_2Ph, H9); 4.11 (H2e, under H_2O signal); 4.70 (d, 1H, CH_2Ph, *J* = 13.69 Hz); 5.26 (d, 1H, H4e, *J* = 13.2 Hz); 5.75 (d, 1H, OH, *J* = 4.77 Hz); 7.77 (tr, 2H, CH(3,5) (C_6H_4F) *J* = 8,56 Hz); 7.84-7.87 (m, 1H, CH(4)(C_4H_3S)), 8.03-8.06 (m, 3H, CH(2,6), (C_6H_4F), CH(3)(C_4H_3S)), 8.48 (d, 1H, CH(5)(C_4H_3S), *J* =4.52 Hz)

NMR ^{13}C (characteristic signals) (DMSO-d_6, δ/ ppm, *J*/Hz): 21.5, 21.7 (both s, $2CH_3$); 36.2, 36.7 (s, C1, C5); 52.7, 57.8 (br.) (all s, C2, C4, C6, C8); 62.4 (s, CH_2Ph); 75.9 (s, C9); 127.3, 128.4, 128.9, 129.0, 130.9, 131.6, 138.0 (all s, Ar); 162,3 (s, C=O).

Found (%): C, 64.89; H, 6.41; N, 6.99. Calculated for $C_{21}H_{25}FN_2O_2S$ (%): C, 64.92; H, 6.49; N, 7.21.

Anti-1,5-dimethyl-3-(2-thiophenecarbonyl)-7-(4-chlorobenzyl)-3,7-diazabicyclo[3.3.1]nonane-9-ol (4be)

70 mg (0.24 mmol) of bispidine **3b** and 34.8 mg (0.24 mmol) of 2-thiophenecarbonyl chloride in 10 ml of dry benzene give gel, from which 0.87 g of the free base was isolated. Yield 90%.

NMR ^1H (DMSO-d_6, δ/ ppm, *J*/Hz): 0.72, 0.79 (both s, 6H, 2CH3); 2.19 (d, 1H, H6e/8e/6a/8a, *J* = 10.39 Hz); 2.24-2.35 (m, 3H, H6e/8e/6a/8a); 2.68 (d, 1H, H4a, *J* = 13.45 Hz); 3.09 (d, 1H, H2a, *J* = 13.45 Hz); 3.12-3.16 (m, 2H, CH_2Ph, H9); 3.41 (d, 1H, H2e, *J* = 12.69 Hz); 3.55 (d, 1H, CH_2Ph, *J* = 13.45 Hz); 4.48 (d, 1H, H4e, *J* = 13.45 Hz); 4,98 (d, 1H, OH, *J* = 5.01 Hz); 7.31-7.31 (m, 6H, CH(Ar)), 7.49(d, 2H, H(2,6)(ArC=O), *J* = 8.19 Hz).

NMR ^{13}C (characteristic signals) (DMSO-d_6, δ/ ppm, *J*/Hz): 21.5, 21.7 (both s, $2CH_3$); 36.2, 36.7 (s, C1, C5); 52.7, 57.5, 57.8 (br.) (all s, C2, C4, C6, C8); 62.4 (s, CH_2Ph); 75.8 (s, C9); 127.3, 128.4, 128.9, 129.0, 130.9, 131.6, 138.0 (all s, Ar); 162,2 (s, C=O).

Found (%): C, 62.21; H, 6.15; N, 6.81. Calculated for $C_{21}H_{25}ClN_2O_2S$ (%): C, 62.28; H, 6.22; N, 6.92.

Anti-1,5-dimethyl-3-(2-thiophenecarbonyl)-7-(4-bromobenzyl)-3,7-diazabicyclo[3.3.1]nonane-9-ol (4de)

140 mg (0.4 mmol) of bispidine **3d** and 60 mg (0.4 mmol) of 2-thiophenecarbonyl chloride in 10 ml of dry benzene give gel, from which 0.149 g of the free base was isolated. Yield 83%.

NMR ^1H (DMSO-d_6, δ/ ppm, *J*/Hz): 0.72, 0.79 (both s, 6H, $2CH_3$); 2.16-2.34 (m, 4H, H6e/8e/6a/8a); 2.66 (d, 1H, H4a, *J* = 12.7 Hz); 3.09 (m, 3H, H2a, CH_2Ph, H9); 3.49 (H2e, under H_2O signal); 3.91 (d, 1H, CH_2Ph, *J* = 12.72 Hz); 4.45 (d, 1H, H4e, *J* = 13.5 Hz), 4,98 (d, 1H, OH, *J* = 4.89 Hz); 7.31-7.31 (m, 6H, CH(Ar)), 7.49(d, 2H, CH(Ar), *J* = 8.19 Hz).

Found (%): C, 56.09; H, 5.77; N, 6.03. Calculated for $C_{21}H_{25}BrN_2O_2$ (%): C, 56.12; H, 5.61; N, 6.23.

4ae*HCl in nitrobenzene

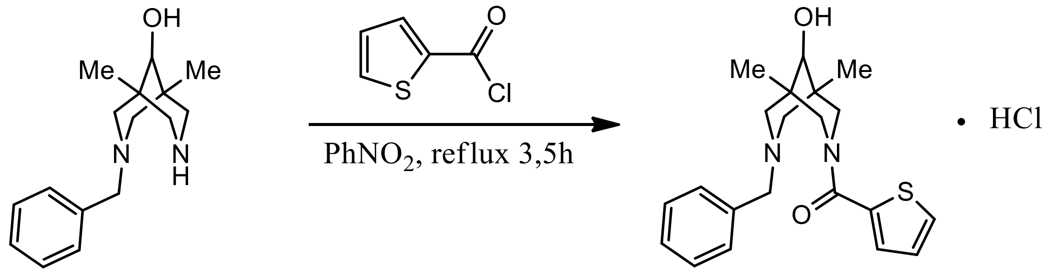

4ae

To a suspension of 70 mg (0.27 mmol) of bispidine **3a** in 2.5 ml of nitrobenzene was added dropwise a solution of 39 mg (0.27 mmol) of 2-thiophenecarbonyl chloride in 2.5 mL of nitrobenzene. Then the mixture was refluxed (210 °C) under vigorous stirring for 3.5 hours. Highly viscous and dense amber gel was formed after 2 days at room temperature.

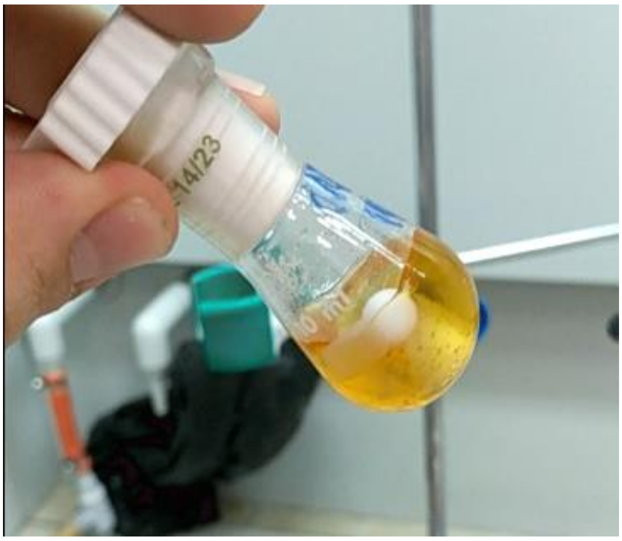

Figure S1. Photo of **4ae*HCl** in nitrobenzene.

4ae*HCl in ethoxybenzene

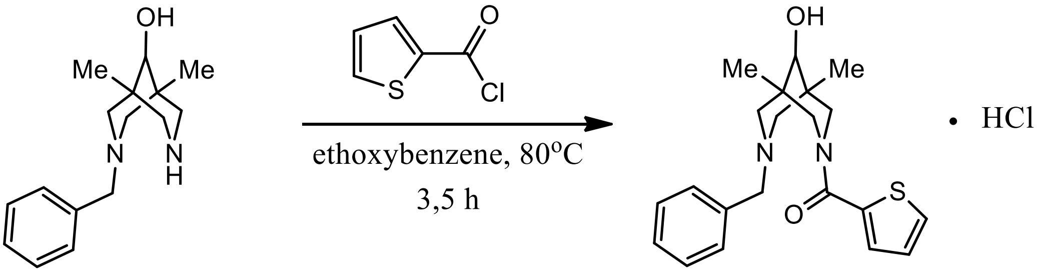

4ae

To a suspension of 70 mg (0.27 mmol) of bispidine **3a** in 2.5 mL of ethoxybenzene was added dropwise a solution of 39 mg (0.27 mmol) of 2-thiophenecarbonyl chloride in 2.5 mL of ethoxybenzene. Then the mixture was stirred at 80 °C for 3.5 hours. Highly viscous and dense colourless gel was formed after 1 hour at room temperature.

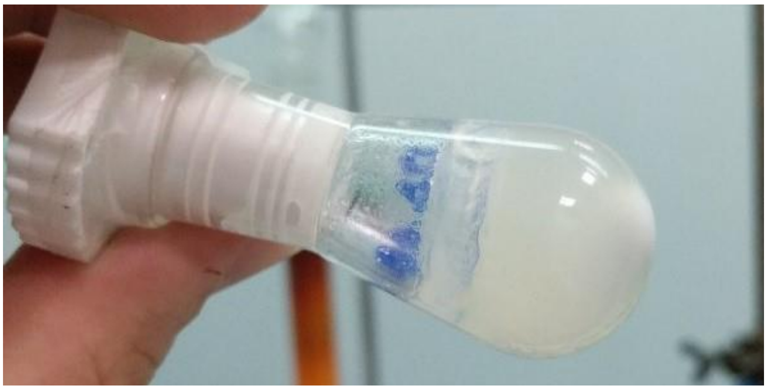

Figure S2. Photo of **4ae*HCl** in ethoxybenzene.

4ae*HCl in mesitylene

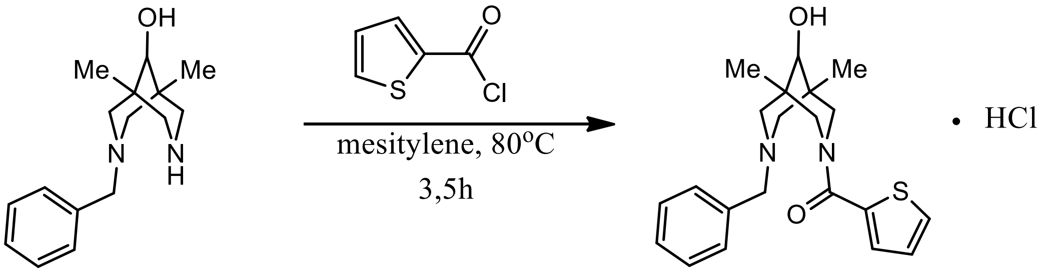

4ae

To a suspension of 70 mg (0.27 mmol) of bispidine **3a** in 2.5 mL of mesitylene was added dropwise a solution of 39 mg (0.27 mmol) of 2-thiophenecarbonyl chloride in 2.5 mL of mesitylene. Then the mixture was stirred at 80 °C for 3.5 hours. Loose colourless gel was formed after 1 hour at room temperature.

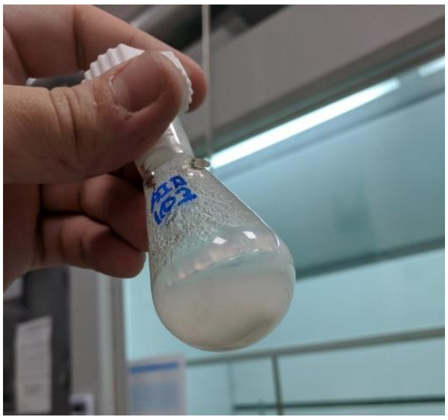

Figure S3. Photo of **4ae*HCl** in mesitylene.

4ab*HCl in nitrobenzene.

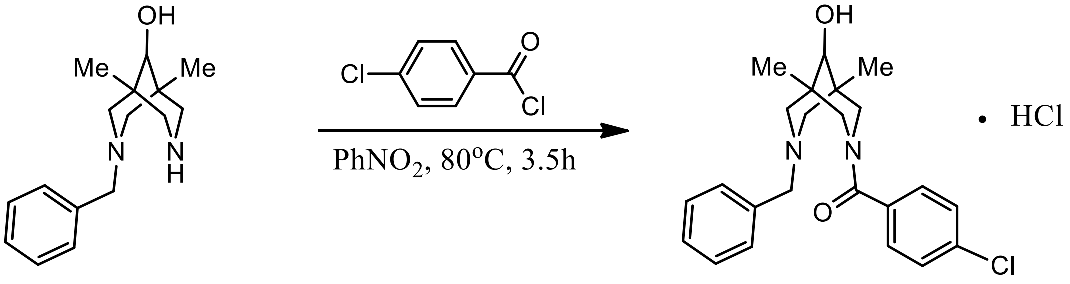

4ab

To a suspension of 70 mg (0.27 mmol) of bispidine **3a** in 2.5 mL of nitrobenzene was added dropwise a solution of 47.1 mg (0.27 mmol) of 4-chlorobenzoyl chloride in 2.5 mL of nitrobenzene. Then the mixture was stirred at 80 °C for 3.5 hours. A precipitate was formed.

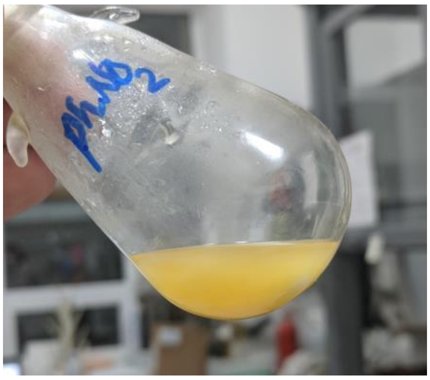

Figure S4. Photo of **4ab*HCl** in nitrobenzene.

4ab*HCl in ethoxybenzene

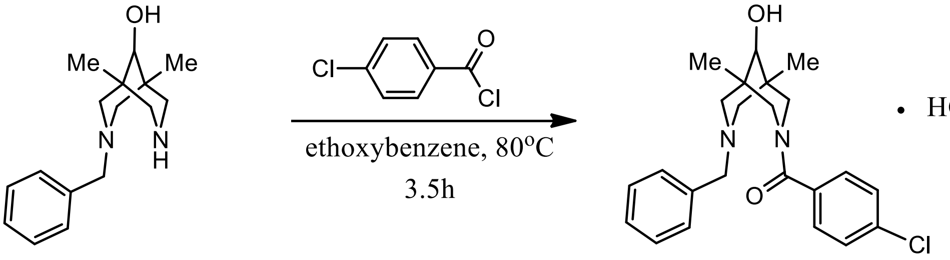

• HCl

4ab

To a suspension of 70 mg (0.27 mmol) of bispidine **3a** in 2.5 mL of ethoxybenzene was added dropwise a solution of 47.1 mg (0.27 mmol) of 4-chlorobenzoyl chloride in 2.5 mL of ethoxybenzene. Then the mixture was stirred at 80 °C for 3.5 hours. Loose colourless gel was formed after 1 hour at room temperature.

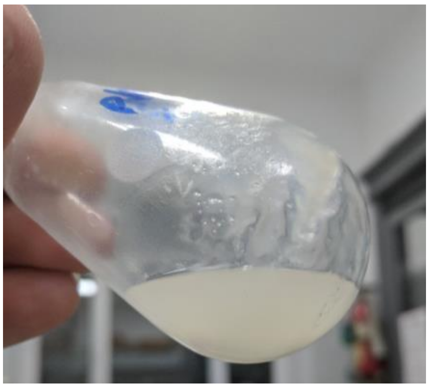

Figure S5. Photo of **4ab*HCl** in ethoxybenzene.

4ab*HCl in mesitylene.

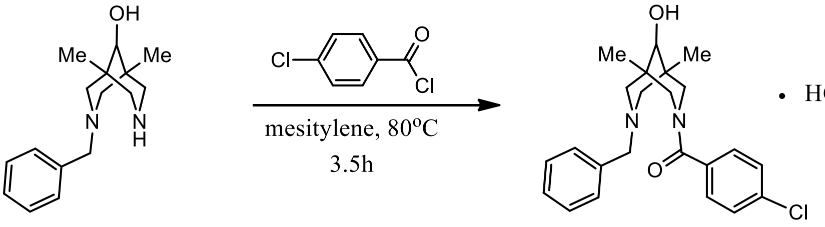

• HCl

4ab

To a suspension of 70 mg (0.27 mmol) of bispidine **3a** in 2.5 mL of mesitylene was added dropwise a solution of 47.1 mg (0.27 mmol) of 4-chlorobenzoyl chloride in 2.5 mL of mesitylene. Then the mixture was stirred at 80 °C for 3.5 hours. Loose colourless gel was formed after 1 hour at room temperature.

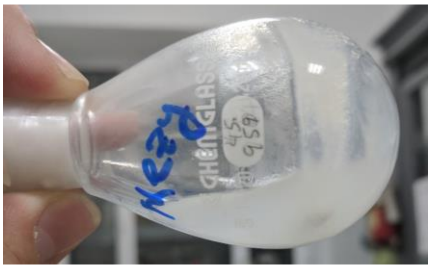

Figure S6. Photo of **4ab*HCl** in mesitylene.

	100×	25×
4cb*HCl		
4ce*HCl		
4bb*HCl		
4bc*HCl		

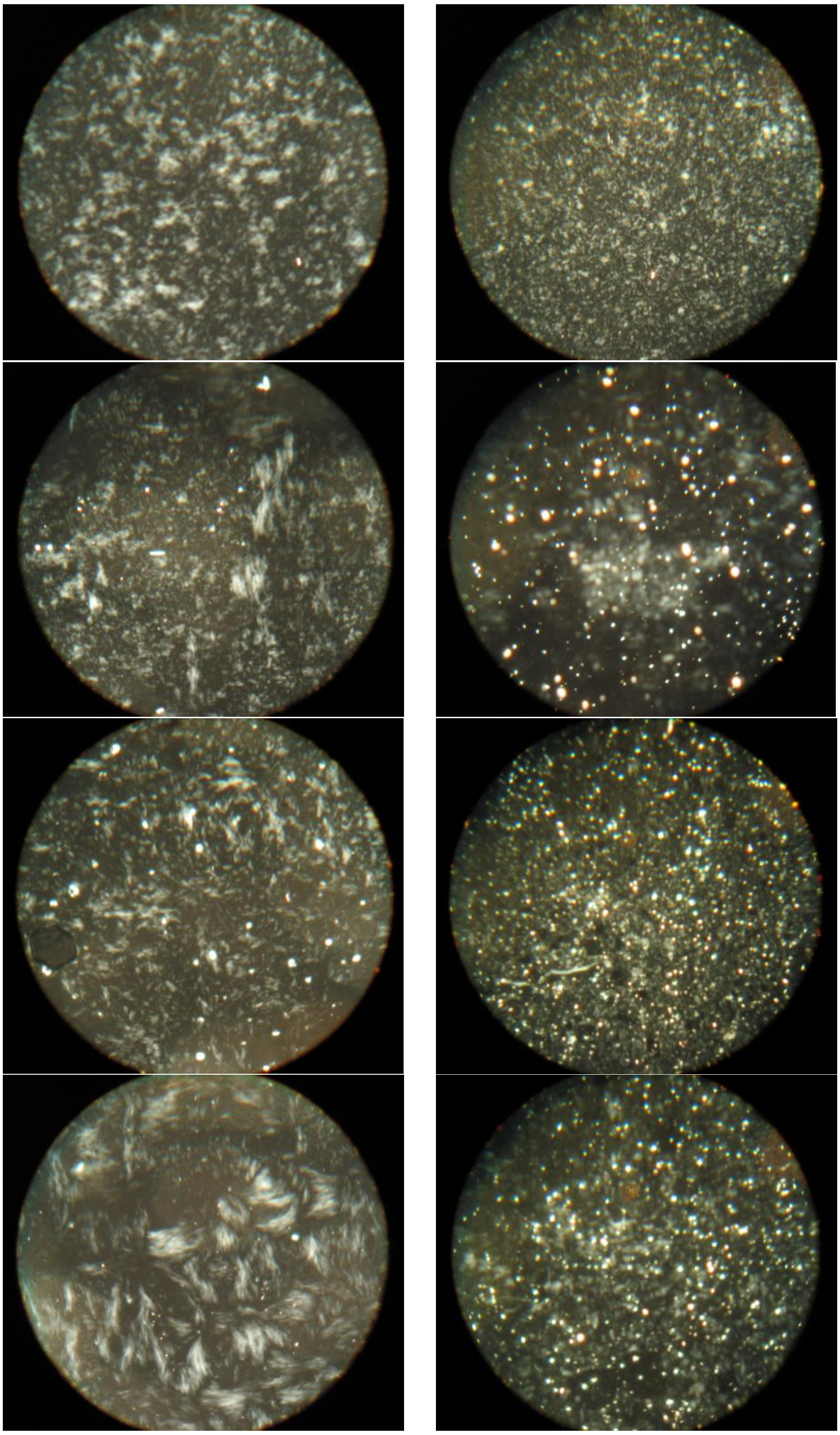

Figure S7. POM photomicrography of benzenogels obtained by Carl Zeiss microscope.

Table S1. Dependence of shear rate on viscosity **benzene@4ce*HCl.**

Shear Rate, Pa	Viscosity, Pa*s
100	0,0975
63,1	0,124
39,8	0,174
25,1	0,246
15,9	0,39
10	0,546
6,31	0,824
3,98	1,46
2,51	1,71
1,59	2,36
1	3,54
0,631	5,81
0,398	9,27
0,251	15
0,158	24,5
0,1	39,1
0,0631	62,2
0,0398	98,6
0,0251	158
0,0158	248
0,01	390
0,00631	613
0,00398	972
0,00251	1520
0,00158	2340
0,001	3680

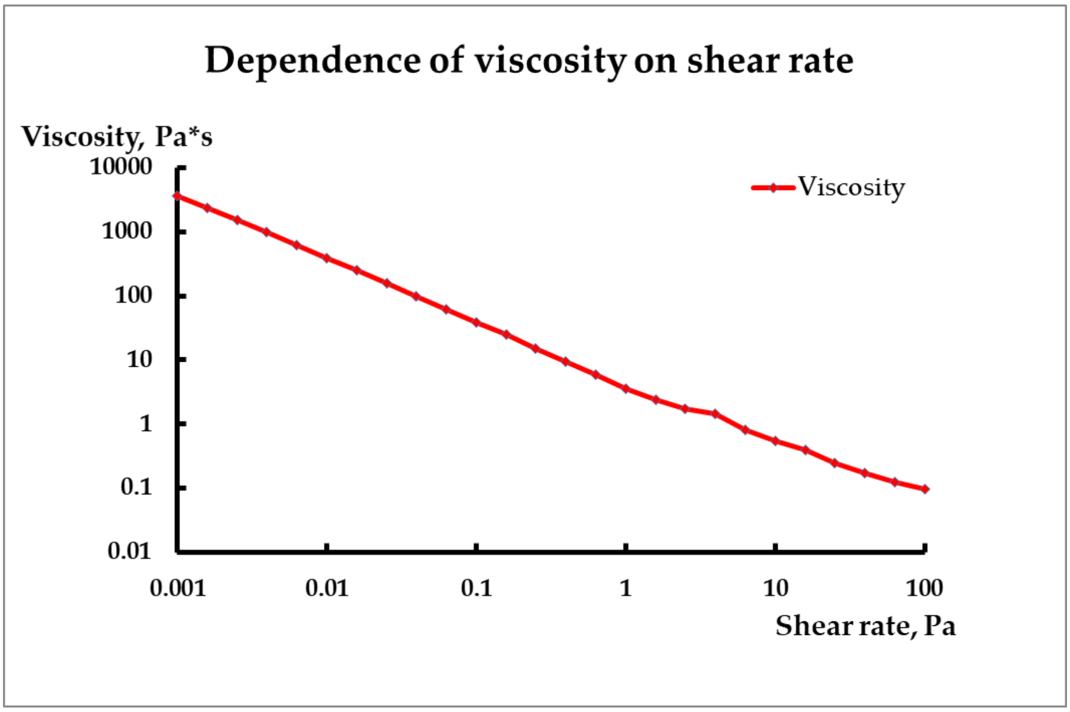

Figure S8. Dependence of viscosity of **benzene@4ce*HCl** on shear rate.

Table S2. Dependence of the loss and storage moduli on angular frequency **benzene@4ce*HCl**.

Angular frequency (ω),Rad/s	Moduli	
	Loss (G”), Pa	Storage (G′), Pa
0,996	56,9	70,1
1,58	119	155
2,5	168	219
3,96	204	278
6,28	246	343
9,96	292	421
15,8	357	516
25	427	599
39,6	535	702
62,8	689	746
99,6	904	851
158	945	934
250	1150	1130
396	1160	1110

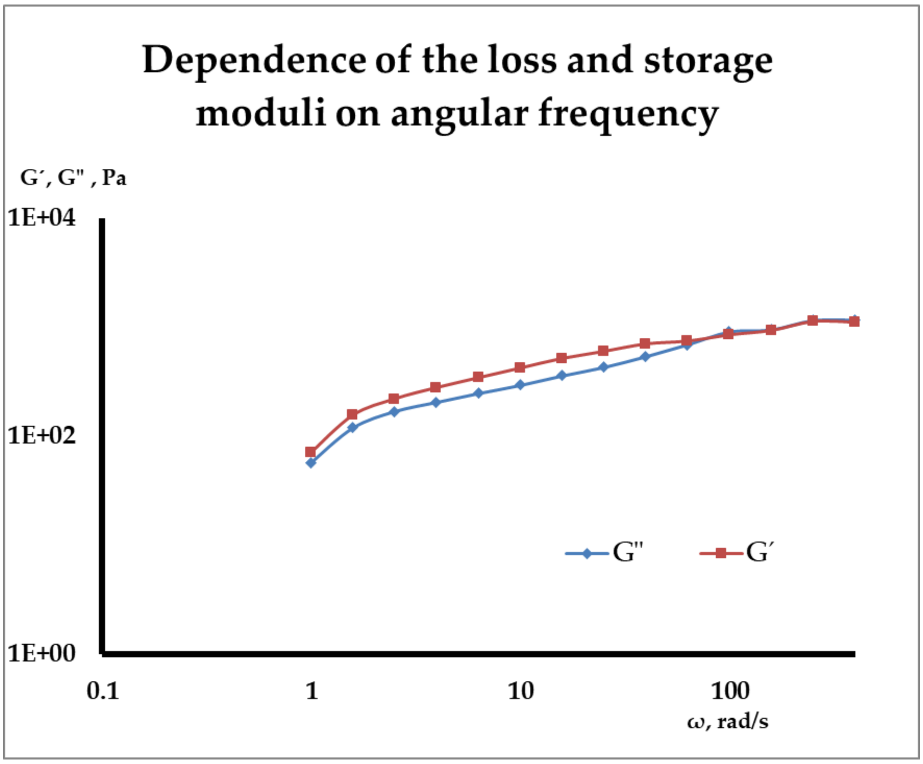

Figure S9. Dependence of the loss and accumulation modules on angular frequency **benzene@4ce*HCl.**

Table S3. Crystal data, data collection, structure solution and refinement parameters for **4cb**, **2c**, **3a**, **3c**, **1b**, **5,7-dimethyl-1,3-diazaadamantan-6-one** and **4da*HCl.**

**Compound**		**4cb**	**2c**	**3a**	**3c**	**1b**	**5,7-dimethyl-1,3-diazaadamantan-6-one**	**4da*HCl*$(C_6H_6)_2$**
Empirical formula		C23H26Cl1F1N2O2	C17H23F1N2O2	C16H24N2O1	C16H23F1N2O1	C34H50Cl2F2N4O5	C10H16N2O1	C29H34Br1Cl1N2O2
Formula weight		416.91	306.37	260.37	278.36	703.68	180.25	557.94
Colour, habit		Colourless block	Colourless block	Colourless block	Colourless prism	Colourless block	Colourless prism	Colourless plate
Crystal size/mm		0.35×0.18×0.08	0.35×0.35×0.20	0.40×0.20×0.10	0.40×0.30×0.20	0.20×0.15×0.06	0.40×0.20×0.10	0.25×0.08×0.01
Crystal system		orthorhombic	orthorhombic	orthorhombic	monoclinic	monoclinic	orthorhombic	monoclinic
Space group		Pna21	Pbcn	Pbca	P21/n	P21	Fdd2	C2/c
Unit cell dimensions:	a/Å	14.4619(10)	19.3091(11)	12.1577(4)	7.8747(5)	7.623(6)	14.7493(16)	27.43(8)
	b/Å	17.2081(12)	8.4233(5)	14.6409(5)	16.6186(10)	14.139(11)	44.868(5)	10.69(3)
	c/Å	8.3953(6)	19.6775(12)	16.6996(6)	12.0211(8)	15.983(13)	5.7913(6)	19.81(5)
	/°	90	90	90	107.649(1)	95.175(13)	90	112.93(4)
Volume/Å3		2089.3(3)	3200.5(3)	2972.52(18)	1499.11(16)	1716(2)	3832.5(7)	5349(26)
Z		4	8	8	4	2	16	8
Density (calculated)/g·cm-3		1.325	1.272	1.164	1.233	1.362	1.250	1.386
Abs. coefficient/mm-1		0.213	0.091	0.073	0.086	0.247	0.082	1.666
F(000)		880	1312	1136	600	748	1568	2320
Temperature/K		120.0(2)	120.0(2)	160(2)	160(2)	150(2)	150(2)	160(2)
range/°		1.84 to 26.00	2.07 to 25.05	2.44 to 28.00	2.16 to 28.00	1.28 to 25.25	1.82 to 30.00	2.07 to 25.25
		-15 h 17	-22 h 23	-15 h 16	-10 h 10	-9 h 9	-20 h 20	-32 h 32
Index ranges		-9 k 21	-9 k 10	-19 k 19	-21 k 21	-16 k 16	-62 k 62	-12 k 12
		-10 l 10	-23 l 13	-22 l 22	-15 l 15	-19 l 19	-6 l 8	-23 l 16
Reflections collected		11202	16298	28966	15258	11580	9106	13547
Unique reflections [Rint]		3955 [Rint=0.0440]	2829 [Rint=0.0437]	3579 [Rint=0.0315]	3617 [Rint=0.0236]	6117 [Rint=0.0903]	1517 [Rint=0.0376]	4817 [Rint=0.235]
Data / restraints / params		3955 / 1 / 269	2829 / 0 / 225	3579 / 0 / 182	3617 / 0 / 273	6117 / 1 / 429	1517 / 1 / 183	4817 / 39 / 295
Reflections with I>2σ(I)		3377	2117	2862	3017	3711	1367	2030
Goodness-of-fit on F2		1.024	1.040	1.085	1.064	0.972	1.074	0.985

Final R indices [I>2σ(I)]	R1 = 0.0418<br>wR2 = 0.0811	R1 = 0.0581<br>wR2 = 0.1198	R1 = 0.0414<br>wR2 = 0.1120	R1 = 0.0399<br>wR2 = 0.1047	R1 = 0.0783<br>wR2 = 0.1699	R1 = 0.0339<br>wR2 = 0.0874	R1 = 0.1159<br>wR2 = 0.2769
R indices (all data)	R1 = 0.0540<br>wR2 = 0.0848	R1 = 0.0810<br>wR2 = 0.1306	R1 = 0.0543<br>wR2 = 0.1188	R1 = 0.0489<br>wR2 = 0.1098	R1 = 0.1320<br>wR2 = 0.1943	R1 = 0.0388<br>wR2 = 0.0904	R1 = 0.2320<br>wR2 = 0.3363
Abs. structure parameter	0.21(7)	—	—	—	0.24(12)	—	—
Largest diff. peak/hole (e·Å-3)	0.170 / -0.180	0.242 / -0.265	0.347 / -0.173	0.360 / -0.200	0.607 / -0.391	0.243 / -0.163	1.413 / -0.682
CCDC deposition number	1838470	1838469	1838468	1838467	1838471	1838466	1838465

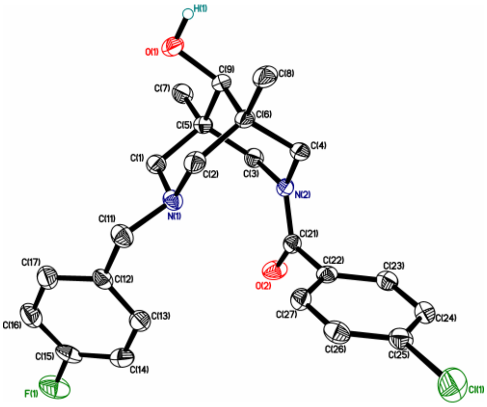

Figure S10. Molecular structure of **4cb**. Displacement ellipsoids are shown at 50% probability level. Hydrogen atoms are omitted for clarity.

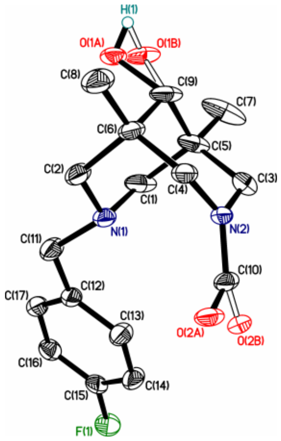

Figure S11. Molecular structure of **2c**. Displacement ellipsoids are shown at 50% probability level. Hydrogen atoms are omitted for clarity. Minor components of disorder are drawn as open lines.

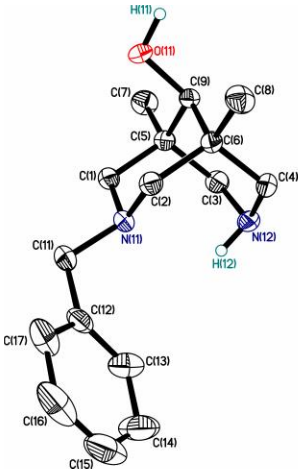

Figure S12. Molecular structure of **3a**. Displacement ellipsoids are shown at 50% probability level. Hydrogen atoms are omitted for clarity.

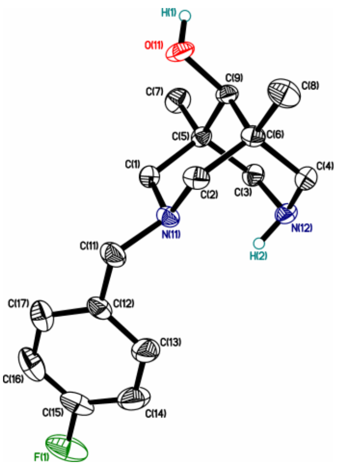

Figure S13. Molecular structure of **3c**. Displacement ellipsoids are shown at 50% probability level. Hydrogen atoms are omitted for clarity.

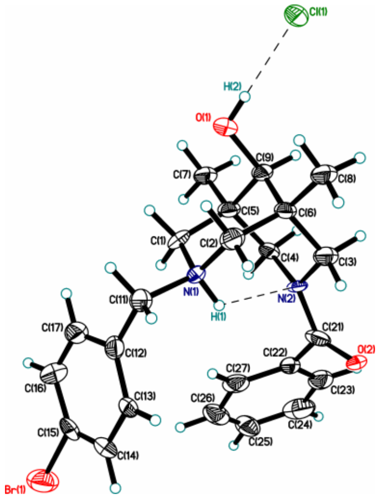

Figure S14. Molecular structure of **4da*HCl*(C_6H_6)$_2$.** Displacement ellipsoids are shown at 50% probability level. Solvent benzen molecules are not shown for cleraty.

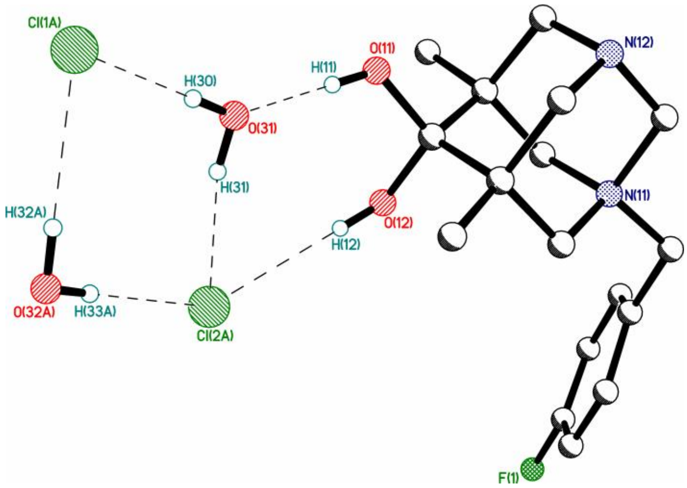

Figure S15. Hydrogen-bonded finite motif in the structure **1c+H2O**.

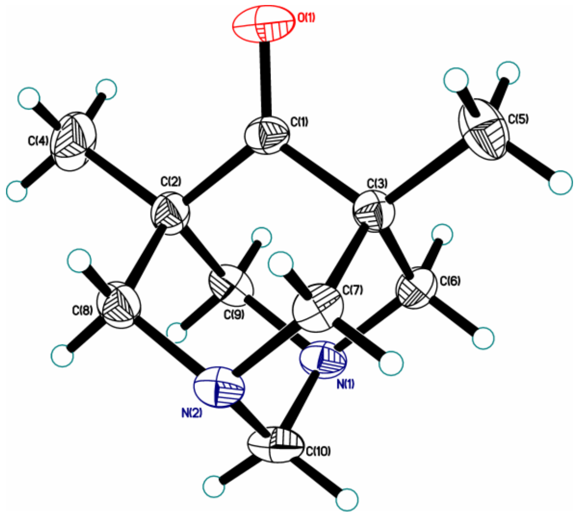

Figure S16. Molecular structure of 5,7-dimethyl-1,3-diazaadamantan-6-one. Displacement ellipsoids are shown at 50% probability level.

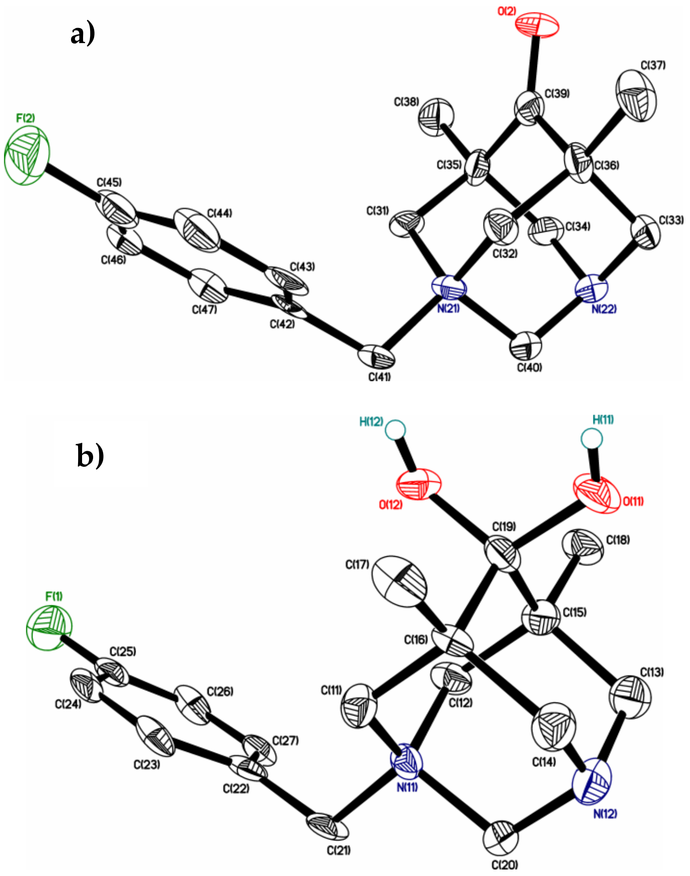

Figure S17. The structures **a)** and **b)** of ketone and its diol form in the crystal **1c**. Displacement ellipsoids are shown at 50% probability level. Hydrogen atoms are omitted for clarity.

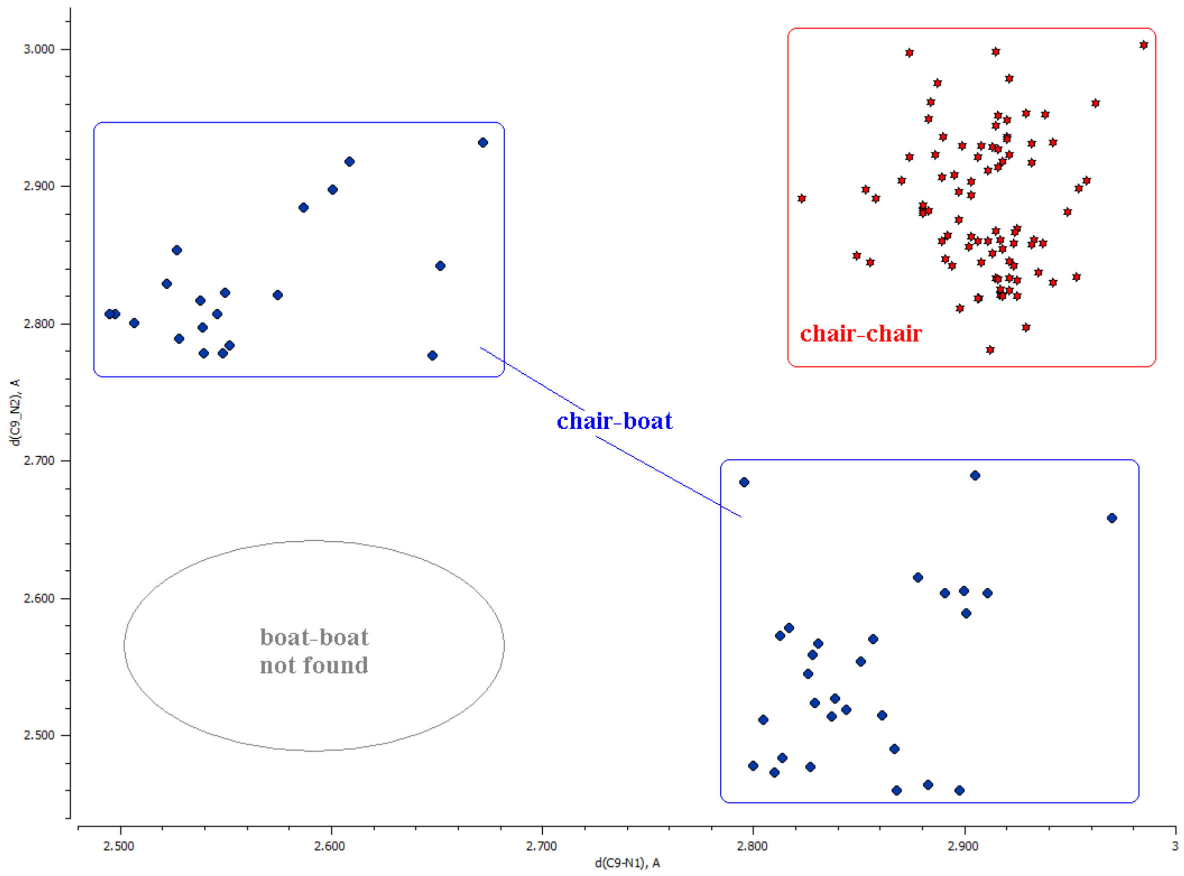

Figure S18. Scatterplot of C9…N separations in structures of neutral organic flexible bispidines.

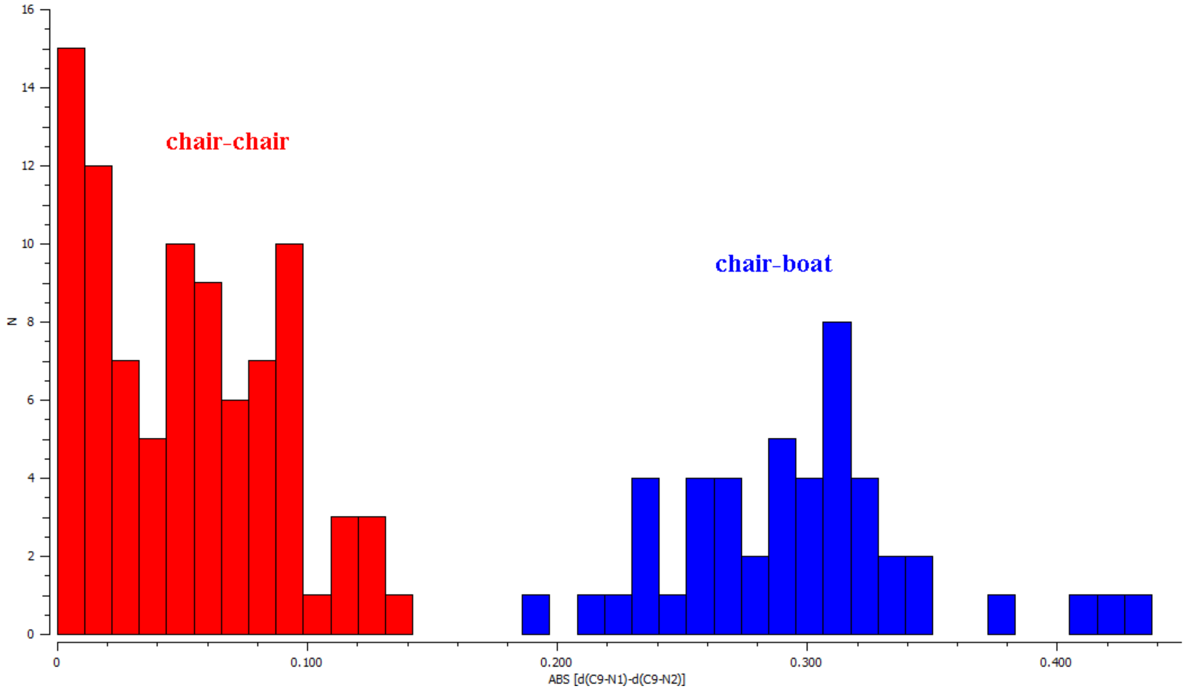

Figure S19. Histogram of absolute differences between C9…N separations.

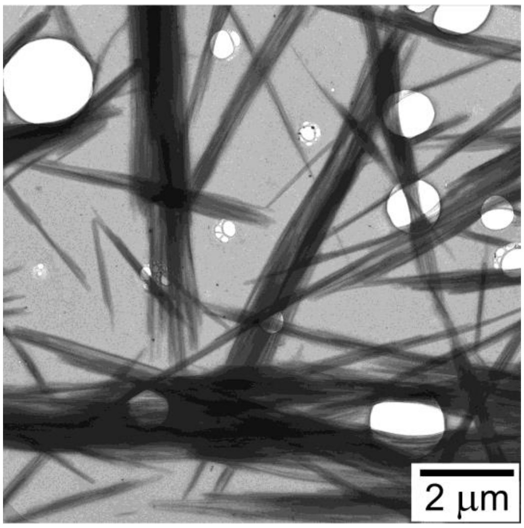

Figure S20. TEM micrograph of **4bc*HCl**.

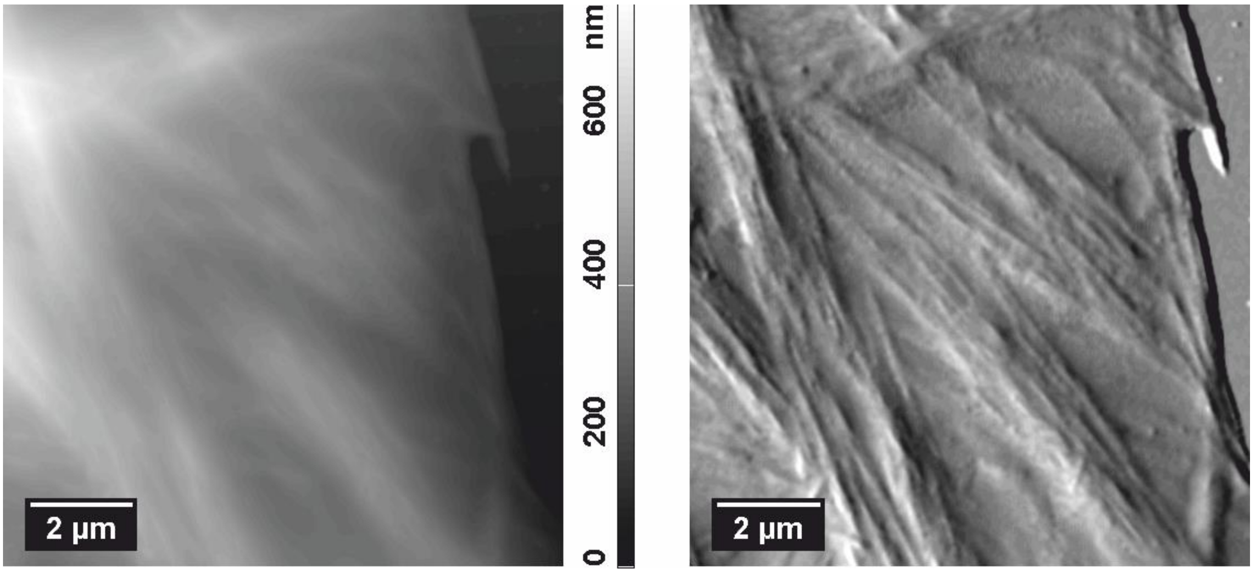

Figure S21. AFM micrograph of **4bc*HCl** (the left picture is topography and the right picture is error signal).

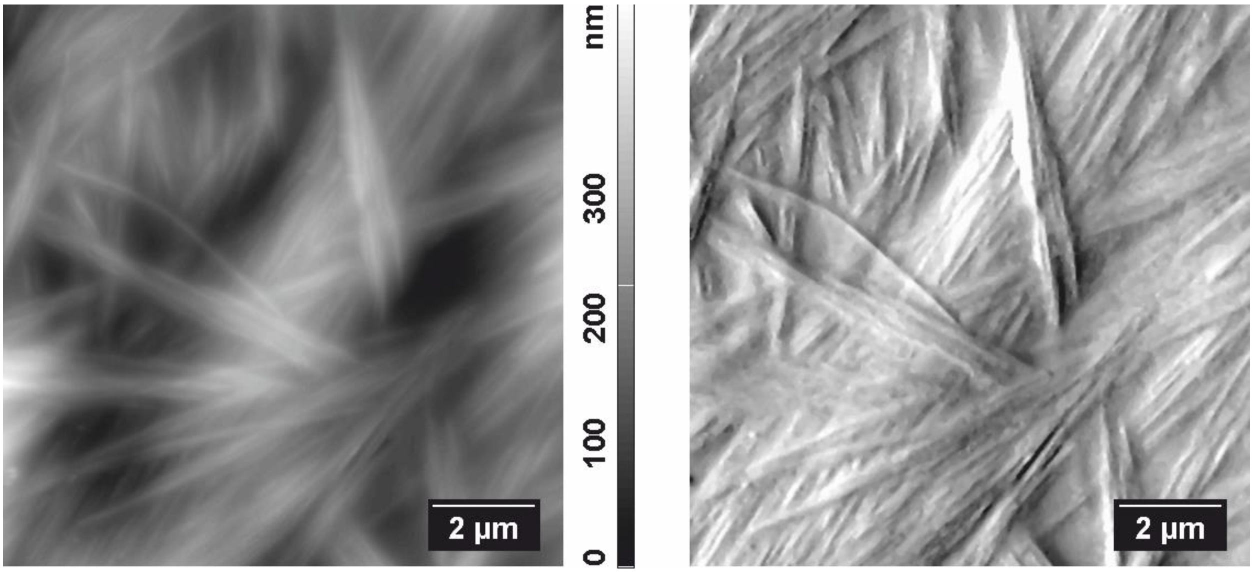

Figure S22. AFM micrograph of **4bc*HCl** (the left picture is topography and the right picture is error signal).

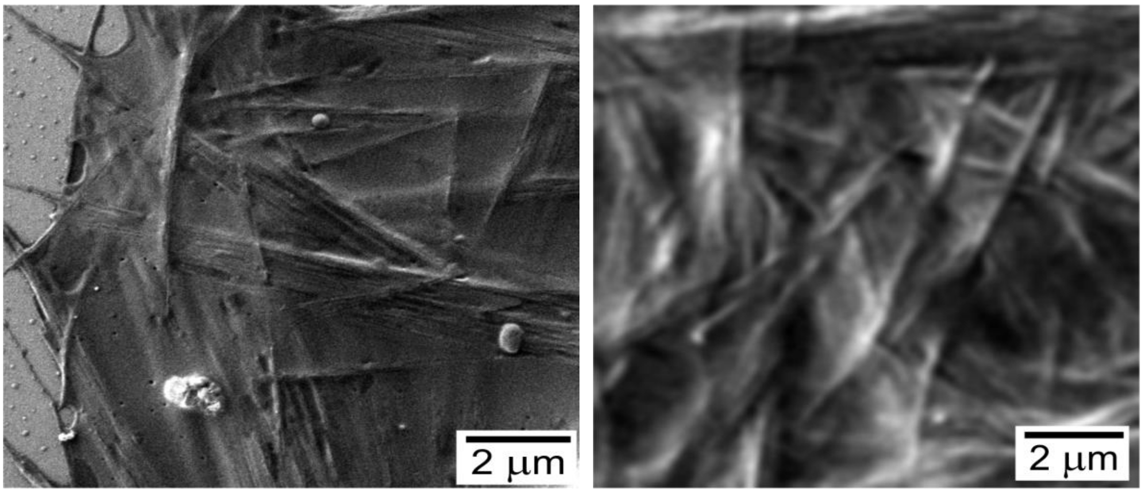

Figure S23. AFM micrograph of **4bc*HCl** (15 K×).

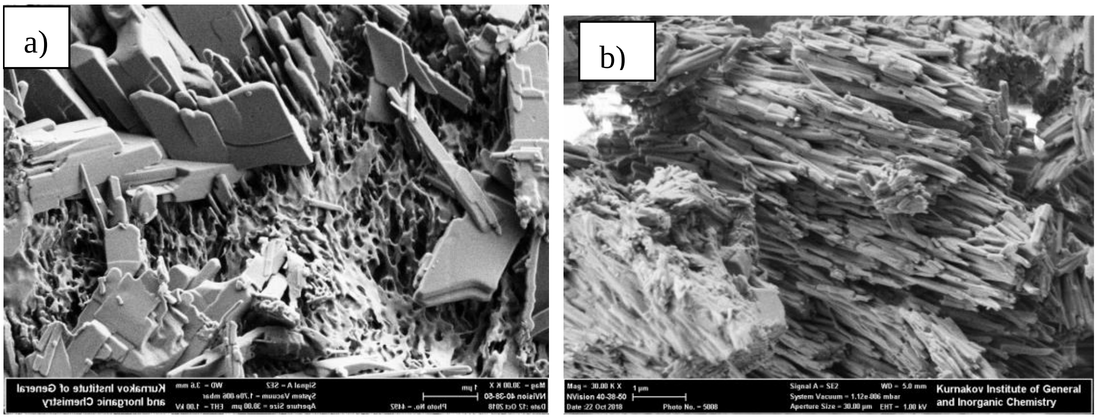

Figure S24. SEM micrographs of dry gel samples made by different methods of solvent removal from **ethoxybenzene@4ae*HCl:** under reduced pressure (**a**), sc-CO2 drying (**b**). Scale bar is 1 µm.

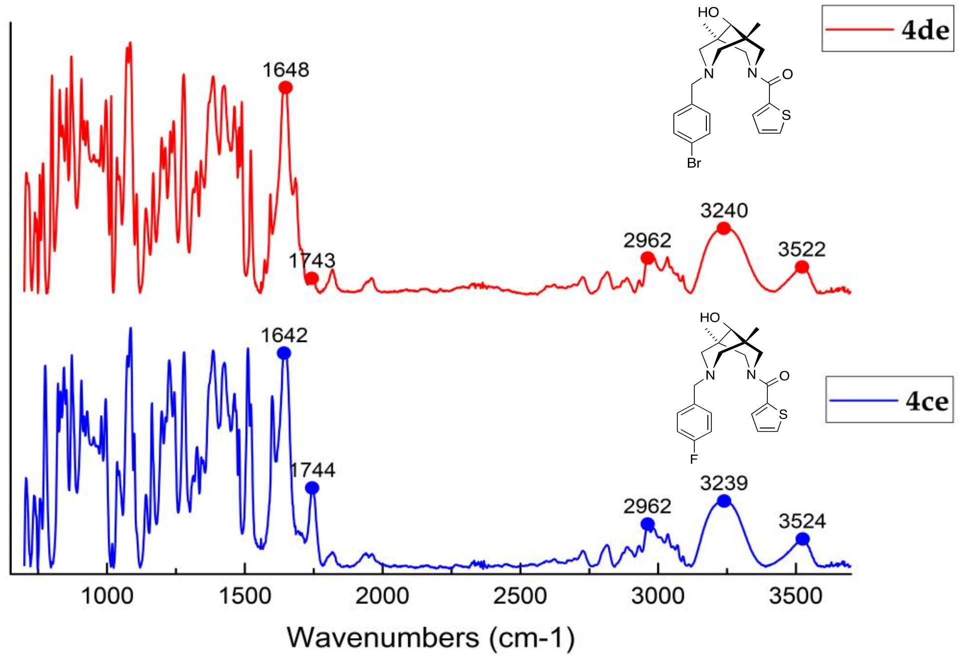

Figure S25. FT IR spectra of the native gels benzene@4de*HCl and benzene@4ce*HCl.
